# Phased ecological assembly of Triassic marine reptile faunas following the Permian–Triassic mass extinction: ecospace dynamics in South China

**DOI:** 10.1098/rspb.2026.2108

**Published:** 2026-10-14

**Authors:** Mingtao Yao, Yinuo Wang, Zhaoliang Ma, Jiandong Huang, Zuoyu Sun

**Affiliations:** ^1^ Department of Geology and Geological Museum, Peking University, Beijing, People's Republic of China; ^2^ Observation and Research Station of Stratigraphy, Paleontology and Environmental Geology in Chaohu, Ministry of Natural Resources, Hefei, People's Republic of China; ^3^ State Key Laboratory of Critical Mineral Research and Exploration, Guiyang, People's Republic of China; ^4^ Department of Research, Anhui Geological Museum, Hefei, People's Republic of China; ^5^ State Key Laboratory of Palaeobiology and Petroleum Stratigraphy, Nanjing Institute of Geology and Palaeontology, Chinese Academy of Sciences, Nanjing, People's Republic of China

**Keywords:** ecospace dynamics, Triassic marine reptiles, ecosystem recovery, P-Tr mass extinction, South China

## Abstract

The Permian-Triassic mass extinction triggered a fundamental reorganization of marine ecosystems, yet the community-level functional dynamics of early marine reptile radiations remain understudied. Here we quantify ecospace dynamics of Triassic marine reptile faunas across an approximately 20-million-year sequence in South China, integrating 10 functional traits of fossil taxa and modern ecological analogues. We identify five ecotypes defined by habitat use, foraging modes and trophic strategy. Statistical analyses reveal cumulative and incremental faunal assembly in South China in three phases: Early Triassic niche conservatism with high functional redundancy; Middle Triassic ecological radiation with expanded disparity and reduced redundancy; and late Middle to Late Triassic ecological transition and ecosystem maturation, culminating in pelagic specialization and megafaunal predation. Environmental amelioration from the late Early Triassic was associated with ecological radiation, while sea-level fluctuations may have contributed to the nearshore-to-offshore transition. Comparison with other Triassic records reveals spatial heterogeneity and temporal asynchrony in ecological assembly: marine reptiles did not occupy roles analogous to those of modern cetaceans in South China until the Ladinian, whereas comparable roles had likely appeared earlier elsewhere. This succession thus records one of several regionally distinct pathways along which marine reptiles acted as ecological innovators during post-extinction recovery.

## 1. Introduction

The Permian–Triassic mass extinction (PTME), the most catastrophic biotic crisis of the Phanerozoic, triggered a fundamental regime shift from Palaeozoic-type to modern-type marine ecosystems [1–3]. In its aftermath, reptiles invaded the marine realm and radiated to become dominant apex predators, refilling vacant niches and driving the reassembly of complex trophic networks [4–6]. As keystone regulators and indicators of ecosystem complexity and stability [7], their ecological evolution serves as a critical proxy for understanding the structural reorganization of post-extinction ecosystems [8,9]. Functionally, Triassic marine reptiles probably occupied ecological roles analogous to those of modern cetaceans, elasmobranchs and coastal crocodylians [10–12], exerting top-down control and enhancing carbon flux pathways within Mesozoic oceans [7,13], and thus providing important deep-time analogues for forecasting potential responses of modern apex species to rapid anthropogenic change [14].

Despite extensive research on post-PTME biodiversity trajectories and environmental fluctuations (e.g. [9,15–18]), the community-level functional dynamics and long-term ecological assembly of marine reptile communities remain understudied [13]. Most previous studies have focused on taxonomic richness or individual lineages, leaving a fundamental gap in our understanding of how functional ecospace was systematically reoccupied and partitioned.

The Triassic fossil Lagerstätten of South China provide an unparalleled temporal window spanning over 20 million years of biotic recovery [4], offering a relatively continuous record of marine reptile community assembly across multiple ecological transitions. This sequence encompasses the Early Triassic Chaohu Fauna [5,19] and Nanzhang-Yuan'an Fauna [20], the early Middle Triassic Panxian Fauna [21] and Luoping Biota [22], the late Middle Triassic Xingyi Fauna [23,24] and the Late Triassic Guanling Biota [25,26].

In this study, we employ a quantitative ecospace framework to characterize the functional evolution of Triassic marine reptile faunas in South China. By integrating 10 multidimensional functional traits, we quantify niche occupation and partitioning analogous to morphospace analyses [27–31]. Large-scale fossil compilations are inherently biased by preservational and sampling heterogeneities, and are inevitably influenced by the patterns of fossiliferous rock availability [32–34]. We instead adopt a focused approach, analysing well-constrained and thoroughly studied fossil Lagerstätten to minimize such biases [35–38]. Specifically, we aim to: (i) reconstruct the ecospace structure of marine reptile faunas and identify distinct ecotypes; (ii) quantify temporal shifts in functional disparity, redundancy and ecospace overlap to delineate evolutionary phases of community assembly; and (iii) evaluate this trajectory against records from elsewhere and contextualize their implications for the post-PTME marine ecosystem recovery.

## 2. Methods

Fossil marine reptile species are independently scored for functional traits and assigned a position within the ecospace. Then they are aggregated by fauna to compare ecospace occupation among the South China Lagerstätten at the community level. The modern tetrapod species included here are not intended to represent a single coherent ecosystem; rather, each is treated as an independent trait-based ecological analogue and is positioned within the same ecospace framework as the fossil taxa to enable direct comparison of ecospace position between fossil ecotypes and their potential modern counterparts.

### (a) Dataset

Our dataset comprises 66 species of Triassic marine reptiles from six exceptionally preserved fossil Lagerstätten of South China [4] (supplementary material, figure S1), alongside 13 species of modern tetrapods with well-documented ecological habits. Modern taxa were selected a priori, independently of the fossil ecotype structure recovered in this study, to represent well-documented ecological strategies spanning a range of present-day marine and semi-aquatic habitats and foraging modes. Triassic marine reptiles belong to six major clades: Ichthyosauriformes, Sauropterygia, Thalattosauriformes, Hupehsuchia, Archosauromorpha and Pantestudines. Ecological and morphological data were compiled from published literature, with scoring criteria following Massare [39,40], Benson *et al.* [41], Dick & Maxwell [30], Foffa *et al.* [14], Reeves *et al.* [31] and Motani & Vermeij [42]. Full taxon lists and ecological scorings are provided in supplementary material.

### (b) Ecospace framework and multivariate ordination

Under the premise of the universal usability in most marine reptilian fossil records, the present ecospace framework consists of 10 major functional traits, following well-established criteria and practices previously used [27,28,30,31], namely diet, hunting style, feeding guilds, feeding selection, habitat, food location, food mobility, aquatic adaptation, armour and body size (table 1; supplementary material). Given the inherent limitations that fossil preservation places on the resolution of ecological data that can be reliably recovered, individual states in this framework are chosen a priori and not customized for particular taxa, such that the framework applied herein matches well with the requirements of a universal theoretical ecospace [28]. Each taxon was coded for either binary or ordinal characters; ordinal coding was applied only where a clear ordering among states existed (table 1).

**Table 1.** Summary of functional traits (bold) and states (numbered) used in the ecospace framework.

traits	states
*binary characters*	
**diet**	1carnivore; 2herbivore
**hunting style**	3ambush; 4pursuit; 5forage
**feeding guilds**	6general; 7cut; 8crunch; 9crush; 10smash; 11pierce; 12lunge; 13filter; 14crush-cut
**feeding selective**	15homodont; 16heterodont
**habitat**	17shallow-coastal water; 18deep open ocean
**food location**	19pelagic; 20benthic
**prey motility**	21free moving; 22sessile; 23non-sessile
**armour**	24non-armoured; 25osteoderms and scutes; 26carapace or plastron only; 27carapace and plastron
**limb shape**	28plesiopedal; 29hydropedal
*ordered, multistate characters*	
30**body length**	(0)<0.5 m; (1)0.5–1 m; (2)1–2 m; (3)2–5 m; (4)5–8 m; (5)>8 m

We performed all analyses using R 4.3.3 [43] and PAST version 4.16c [44]. The analytical workflow commenced with the Triassic fossil-exclusive dataset that comprises binary and ordinal trait scores. It was first transformed into a pairwise inter-taxon dissimilarity matrix through Gower's similarity coefficient [45], which is implemented via the *cluster* package for R [46]. Gower's similarity was prioritized because, compared with other distance estimation methods, it handles mixed data types effectively through variable standardization and mitigates bias from missing values [47,48]. The small number of missing values (2.07% of the total scores) was automatically addressed via pairwise deletion during matrix computation.

The resulting similarity matrix was subsequently ordinated using non-metric multidimensional scaling (NMDS) in the *vegan* package [49], and the maximum dimensionality of the ecospace was set to two-dimensional (2D) and three-dimensional (3D) configurations. The performance of the NMDS was evaluated using stress values, with convex hull polygons used to delineate ecospace occupation areas for comparative faunal analyses. Stress values quantify the discrepancy between the original dissimilarities and distances in reduced-dimensional ordination space; lower stress indicates better preservation of the original data structure, with values <0.2 considered acceptable [50,51]. Hierarchical clustering analysis (with bootstrap resampling, *n* = 5000) was conducted in PAST version 4.16c [44] to quantify overall differences in eco-functional structure based on Gower's distance. This workflow was subsequently conducted for the complete dataset that integrates fossil marine reptiles and modern tetrapods, providing a further robustness check on the fossil-derived ecotype structure.

### (c) Ecological niche metrics and functional diversity analysis

Taxa were binned by ecological (e.g. fauna/dental guild) and evolutionary (e.g. clade/temporal bin) criteria to quantify ecological niche dynamics through functional diversity, redundancy, disparity and ecospace overlap.

Functional *α*-diversity was quantified through ecospace occupancy (convex hull volumetry) and the number of functional entities (counts of unique trait combinations). To statistically evaluate whether ecospace occupation significantly overlaps, non-parametric multivariate analysis of variance (NP-MANOVA) was implemented in the R package *vegan* [49] (supplementary material). The null hypothesis (H₀) posits identical centroid positions across groups in ecospace. H₀ was rejected at the significance level = 0.05 when between-group dispersion exceeded within-group variance. Significant deviations from H₀ indicate that the groups in question occupy ecospace areas that are significantly different. Changes in ecospace occupation over time, if confirmed by NP-MANOVA outcomes, were interpreted as evidence for the migration of marine reptiles into different niche space.

Functional redundancy in each fauna was quantified using mean nearest neighbour distance (MNND) and standard deviation of nearest neighbour distance (SDNND), which characterize the density, aggregation and evenness of species in ecospace. Potential spatiotemporal shifts in niche occupation intensity were visualized through highest density regions generated via Gaussian kernel smoothing in the R package *ggdensity* [52] (supplementary material).

To disentangle the contributions of subgroups (e.g. clade and dental guild) to functional disparity (FD) of each fauna group that contains the corresponding subgroups, partial disparity (PD) indices were adapted from the approach of calculating morphological disparity [53]. PD was calculated as the average squared distance of the point of each subgroup member from the overall group centroid. The resulting value was weighted by a factor that takes into account the sample size of the subgroup compared with the overall sample. That is, *FD* = $\underset{i}{\sum }\underset{j}{\sum }d_{ij}^{2}/(N-1)$, *PD* = $\underset{j}{\sum }d_{ij}^{2}/(N-1)$, where $n_{i}$ is the number of points in subgroup *i*, $N\;=\;\sum n_{i}$ and $d_{ij}^{2}$ is the squared distance of the *j-*th point in subgroup *i* from the overall centroid.

## 3. Results

### (a) Composition of ecospace

NMDS ordination of the fossil dataset placed all examined fossil species into an ecospace, visualizing how reptilian communities cluster together by ecological traits (figure 1). The distance between points within the ecospace reflects the dissimilarity in ecological niches; the farther apart the points, the greater the differences in their niches and the more distinct their ecological functions within the community.

**Figure 1.**
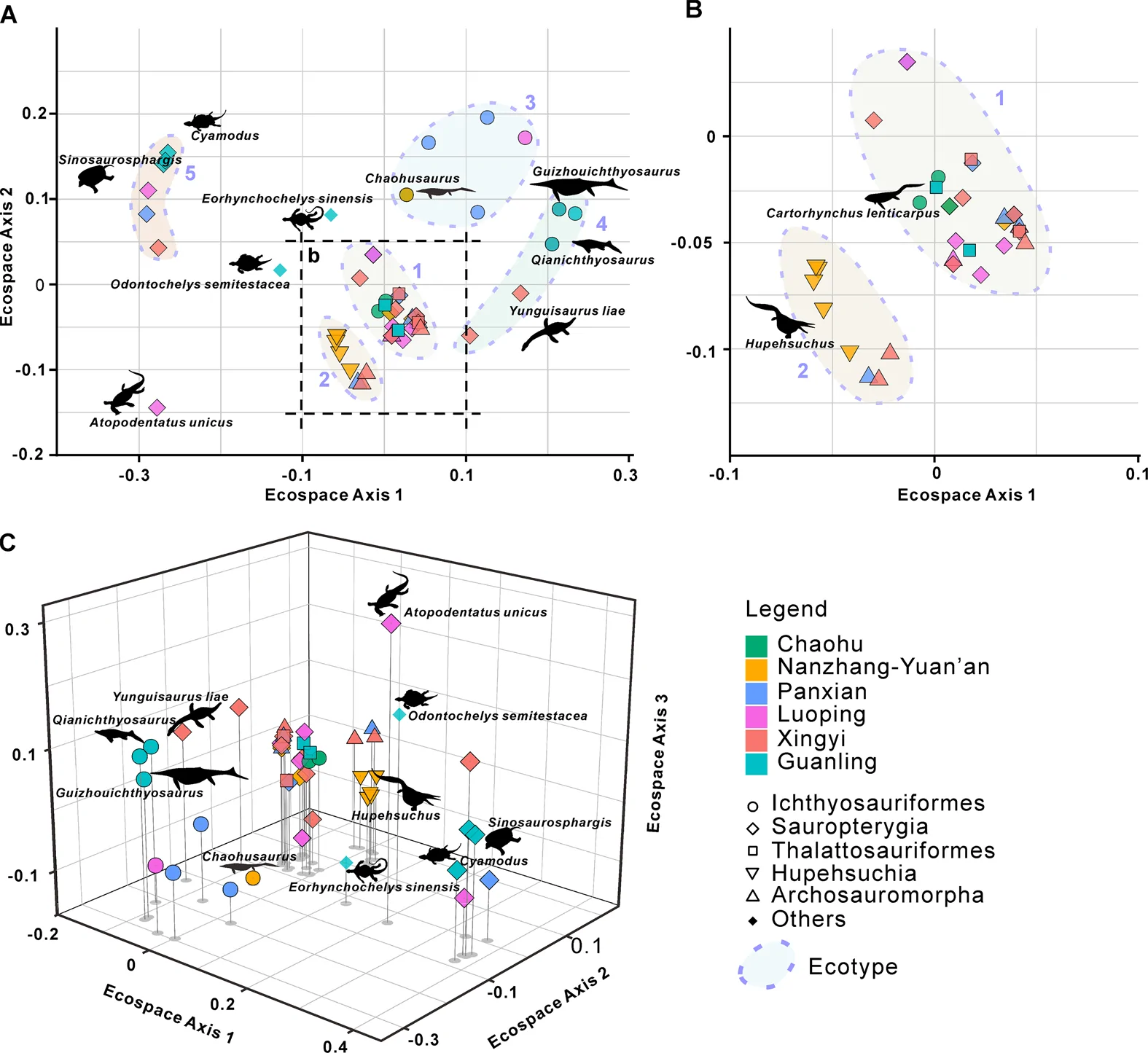
NMDS ordination of Triassic marine reptile ecospace occupation in the South China Basin. (A) Bivariate NMDS projection demonstrating clustered ecospace partitioned into five ecotypes. (B) Magnified view of the densest area (dashed rectangle in A). (C) Three-dimensional NMDS projection showing ecospace occupation. Taxon silhouettes were obtained under Creative Commons licenses from PhyloPic (http://www.phylopic.org/). Stress values: 0.1152 (2D) and 0.0794 (3D).

The stress values for the NMDS were 0.1152 for the two-dimensional set and 0.0794 for the three-dimensional set, respectively (supplementary material, figure S2), indicating that the original data structure is well preserved [50]. Pairwise comparisons revealed a significant divergence (supplementary material, table S1; NP-MANOVA, *p* < 0.001) between all ecotype pairs, further underscoring the distinctiveness of each ecotype in the ecospace. A total of five ecotypes of species, with the exception of some isolated species with distinct combinations of ecological traits, are separated within the ecospace of 2D NMDS (figure 1A,B). These results are validated by the hierarchical clustering results (supplementary material, figure S3). The examination of ecological character distributions (supplementary material, figure S4) reveals that characters such as foraging mode, habitat, limb shape and armour type play a great role in the spatial distribution of taxa within the ecospace, consistent with the results reported for Mesozoic marine tetrapods more broadly [31]. Repeated 3D NMDS recovered no additional discernible structure (figure 1C).

**Ecotype 1**, the most densely populated region, comprises taxa that inhabit shallow coastal water environments, adopt ambush hunting strategies, and feed on pelagic prey. The swimming ability of marine reptiles in this group is not highly developed, with most taxa having plesiopedal limbs. This group includes species of nothosaurs, pachypleurosaurs, the most basal ichthyosauriforms (Nasorostra), all thalattosaurs and some archosauromorphs. Though this group comprises the greatest number of species, their body sizes generally range from size category < 0.5 m to 1–2 m, with the exception of very few ones that reach 2–5 m; their dental guilds are diverse, including general, pierce, crush, smash, crunch and filter types (supplementary material, figure S4). **Ecotype 2** also represents ambush carnivores that feed on pelagic prey and share most ecological characteristics with those of group 1. However, taxa in this group are represented by Hupehsuchia and some archosauromorphs armoured with osteoderms and scutes. **Ecotype 3**, differentiated from the first two groups by taxa with hydropedal limbs that enhance their swimming abilities and aquatic adaptations, can be further subdivided into two subsets: subset 1 includes basal ichthyosauriforms that primarily occupy nearshore shallow environments, while subset 2 contains more derived ichthyosauriforms that display some capacity for deep open water. The dental guilds include crunch, crush and smash types. **Ecotype 4** is characterized by pursuit carnivorous behaviours that target pelagic prey in open-sea environments, and exhibits hydropedal limbs and larger body sizes, with the sole species exceeding 8 m in size. This group includes pistosaurs and derived ichthyosauriforms. **Ecotype 5** comprises partially and fully armoured foragers with plesiopedal limbs that inhabit shallow water and feed on benthic prey. This group contains placodonts and saurosphargids that belong to the crush guild. Isolated within the ecospace, ecological outliers include the enigmatic herbivore *Atopodentatus* and primitive turtles that occupy unique trait combinations (supplementary material, figure S4). During the Early Triassic, three foundational ecotypes of marine reptiles emerged as dominant: ecotype 1 and ecotype 3 were shared between the Chaohu and Nanzhang-Yuan'an faunas, while ecotype 2 was restricted to the latter. The early Middle Triassic (Anisian) witnessed increasing ecological innovations of marine reptiles with the emergence of ecotype 5 in both the Panxian Fauna and Luoping Biota, alongside the herbivorous *Atopodentatus,* a taxon unique to the Luoping Biota [54]. From the late Middle Triassic (Ladinian) to the early Late Triassic (Carnian), represented by the Xingyi Fauna and Guanling Biota, ecological niche partitioning of marine reptiles underwent further expansion, culminating in the ascendancy of ecotype 4. This ecotype included large oceanic predators such as *Guizhouichthyosaurus* spp. and *Guanlingsaurus liangae*, which flourished in open-ocean habitats [26,55].

The iteration of ecospace, as shown by the NMDS results of the dataset that comprises both fossil marine reptiles and their potential ecological analogues (figure 2), is consistent with the ecotypes that were derived from the fossil-exclusive dataset (figure 1). Stress values for the NMDS were 0.1303 for the 2D set and 0.0837 for the 3D set (supplementary material, figure S2), respectively, indicating that the results fit the data well [50]. In both the 2D and 3D NMDS plots (figure 2), *Physeter macrocephalus*, *Orcinus orca* and *Stenella attenuata* were consistently positioned within ecotype 4, quite closely aligned with the large pelagic ichthyosaurs from the Late Ladinian Xingyi Fauna and the Early Carnian Guanling Biota. Meanwhile, *Zalophus californianus*, *Callorhinus ursinus* and *Mirounga angustirostris* were positioned within ecotype 3, quite closely aligned with the early members of Ichthyosauriformes. *Crocodylus porosus*, which represents shallow-coastal, armoured ambush carnivores, was positioned near ecotype 2. *Amblyrhynchus cristatus* showed a close proximity to *Atopodentatus* in 2D NMDS; however, this spatial relationship was not pronounced in 3D NMDS. By contrast, other marine mammals (e.g. *Odobenus rosmarus*) and turtles (e.g. *Chelonia mydas* and *Lepidochelys kempii*) were situated at a relatively distant position from all the Triassic marine reptiles.

**Figure 2.**
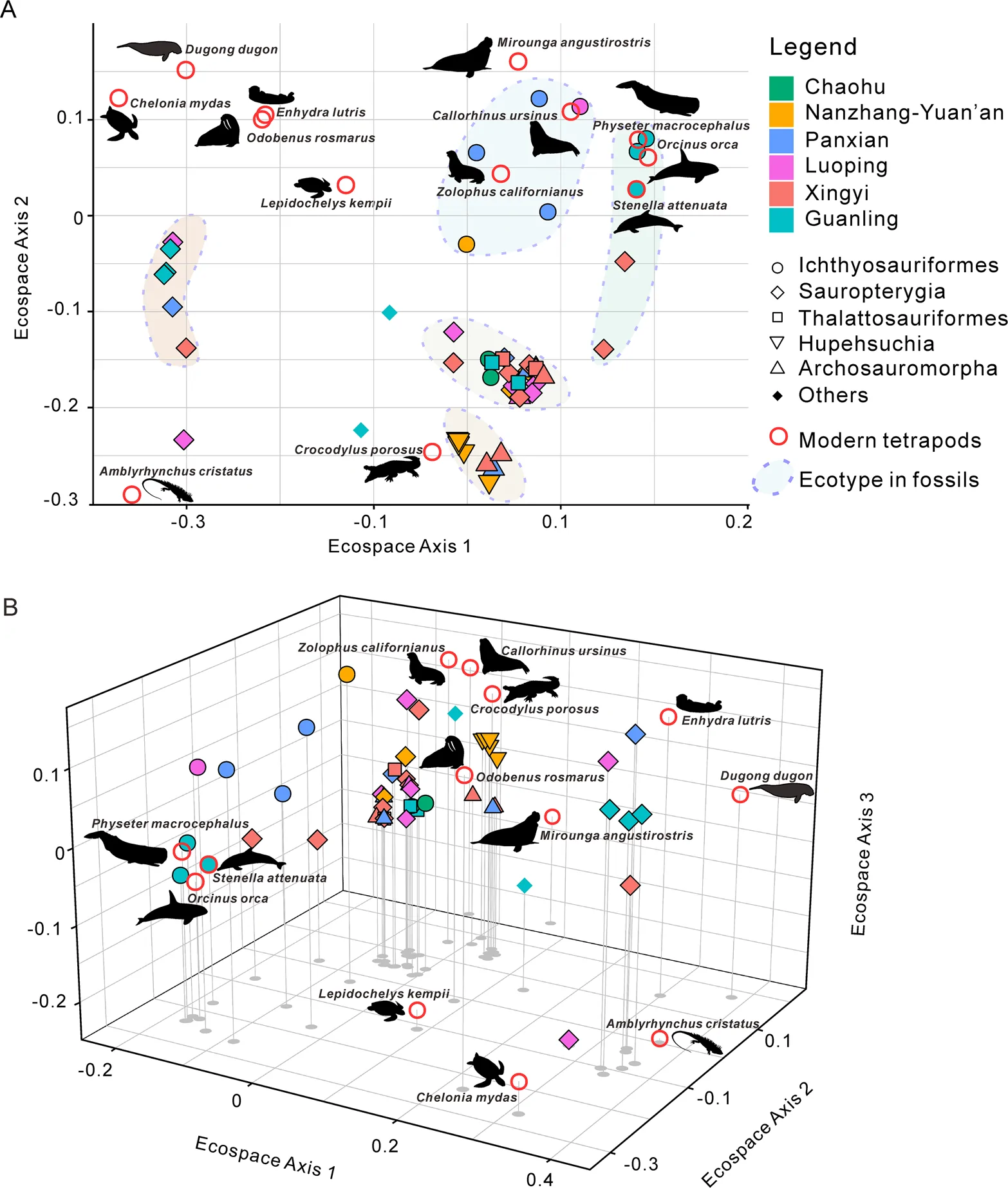
NMDS ordination based on a dataset integrating both fossil and representative modern taxa. (A) Bivariate NMDS projection showing ecospace occupation. (B) Three-dimensional NMDS projection showing ecospace occupation. Taxon silhouettes were obtained under Creative Commons licenses from PhyloPic (http://www.phylopic.org/). Stress values: 0.1303 (2D) and 0.0837 (3D).

### (b) Faunal ecospace occupancy and faunal ecospace overlap

While the number of functional entities does not strictly correlate with species richness, the two diversity curves exhibit broadly consistent trajectories (figure 3A). However, temporal trends in ecospace occupancy are decoupled from diversity patterns. The Olenekian Chaohu Fauna and the slightly younger Nanzhang-Yuan'an Fauna are characterized by limited ecospace occupation and low functional entity counts relative to other younger faunas (figure 3A,B). During the Anisian, both the Panxian Fauna and Luoping Biota display marked increases in ecospace occupation. The Luoping Biota (Anisian) achieved the maximal ecospace occupancy despite reduced species richness and functional entity counts compared with its temporal neighbours—the Anisian Panxian Fauna and the Ladinian Xingyi Fauna. The Ladinian Xingyi Fauna and the Carnian Guanling Biota occupy ecospace areas of comparable magnitude, yet both were smaller than those of the Anisian Panxian Fauna and the Luoping Biota. Notably, the Ladinian Xingyi Fauna, while exhibiting a marginal contraction in convex hull area relative to Anisian predecessors, represents the peak in both species diversity and functional entity richness among all examined marine reptile faunas (figure 3A,B).

**Figure 3.**
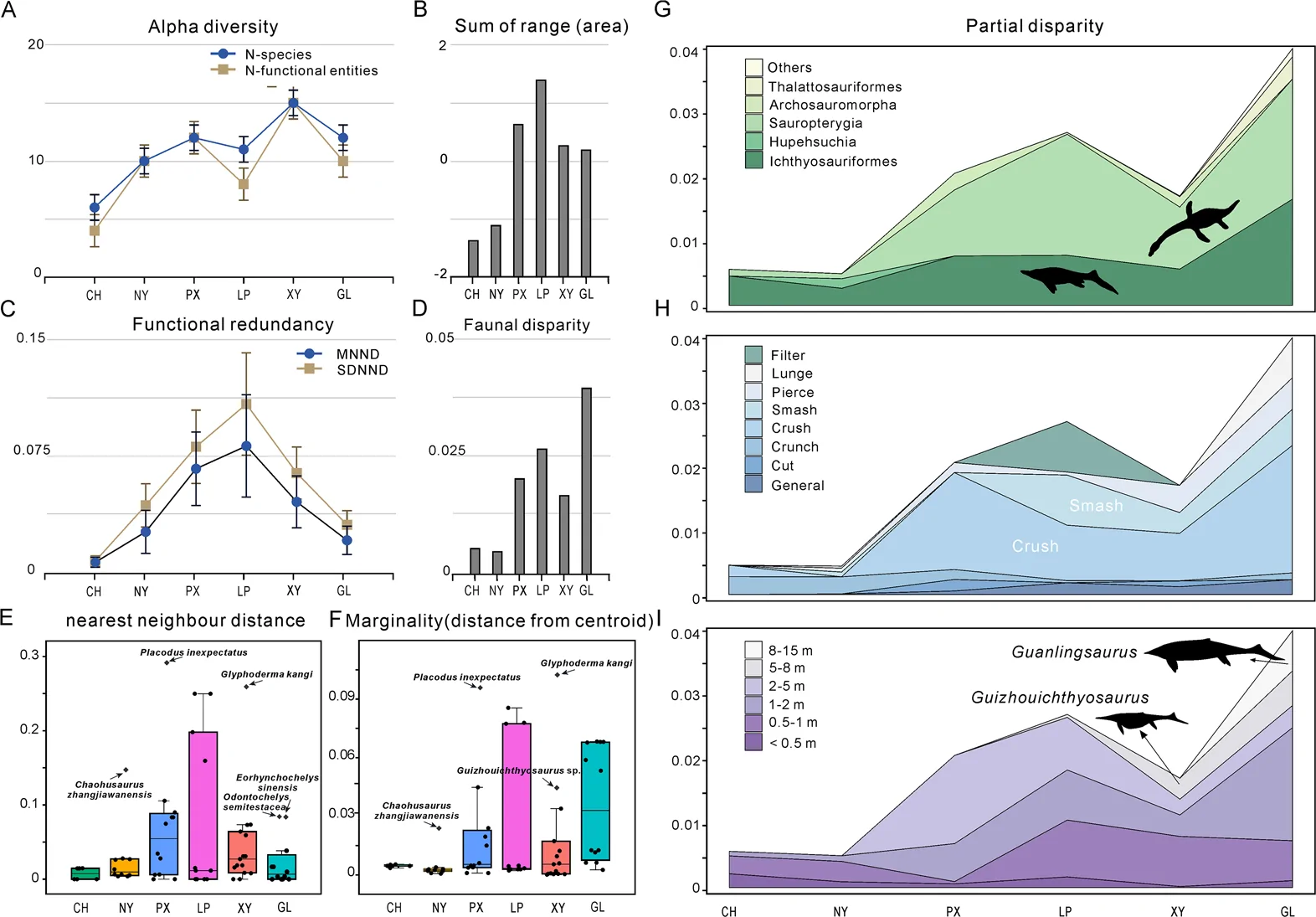
Quantitative characterization of ecological niche dynamics in Triassic marine reptile faunas. (A) Faunal *α*-diversity expressed as species richness and functional entities, error bars = ±1 s.e. (B) Faunal ecospace occupation indicated by the sum of range (area) in 2D NMDS ecospace. (C) Functional redundancy indicated by the values of mean nearest neighbour distance (MNND) and standard deviation of nearest neighbour distance (SDNND), error bars = ±1 s.d. (D) Faunal disparity of six marine reptile faunas. (E) A box plot showing the nearest neighbour distance of each taxon within each fauna, with outliers marked. (F) A box plot showing the marginality of taxa in each fauna. (G–I) Partial disparity of Triassic marine reptile faunas from South China. (G) Partial disparity of taxonomic groups. (H) Partial disparity of dental guilds. (I) Partial disparity of groups divided by body size. Abbreviations: Chaohu (CH), Nanzhang-Yuan'an (NY), Panxian (PX), Luoping Biota (LP), Xingyi (XY), and Guanling Biota (GL).

NP-MANOVA was applied to evaluate the statistical significance of ecospace overlap among those six marine reptile faunas based on both 2D and 3D NMDS configurations (supplementary material). Pairwise comparisons in the 2D NMDS analysis revealed a significant ecospace divergence solely between the Olenekian Chaohu Fauna and the slightly younger Nanzhang-Yuan'an Fauna (*p* = 0.0267). By contrast, the inclusion of a third dimension in NMDS enhances sensitivity to ecological partitioning. The 3D NMDS results identified additional significant statistical differences; the Nanzhang-Yuan'an Fauna exhibited a pronounced divergence from the Ladinian Xingyi Fauna (*p* = 0.0302) and the Carnian Guanling Biota (*p* = 0.0025), while the Chaohu Fauna also showed marginal separation from the Ladinian Xingyi Fauna and the Carnian Guanling Biota (*p* = 0.0638 and *p* = 0.0574, respectively). The Carnian Guanling Biota demonstrated the strongest dissimilarity in ecospace structure within the 3D NMDS, as evidenced by its highly significant divergence from the Nanzhang-Yuan'an Fauna (*p* < 0.001).

### (c) Functional redundancy

Both MNND and SDNND exhibited a unimodal trajectory, with values rising from the Olenekian Chaohu Fauna to a peak in the Anisian Luoping Biota, followed by a subsequent decline (figure 3C). Lower MNND and SDNND correspond to higher density and evenness within communities, which are indicative of elevated functional redundancy. Specifically, functional redundancy, quantified here as the spatial clustering and trait overlap of species of ecological analogues, decreased as MNND and SDNND increased towards their maxima, then rebounded as these metrics declined. This trend was also corroborated by the distribution of nearest neighbour distance (figure 3E) and marginality (distance from faunal centroid) (figure 3F).

### (d) Faunal disparity and partial disparity

During the early intervals, the Olenekian Chaohu Fauna and Nanzhang-Yuan'an Fauna displayed a relatively low faunal disparity. A marked rise in faunal disparity occurred in the Anisian Panxian Fauna and the Luoping Biota. This trend continued into the Ladinian and Carnian stages, with the Guanling Biota demonstrating the highest disparity value (figure 3D).

Partial disparity was calculated for subgroups that were divided by clades (figure 3G), dental guilds (figure 3H) and body sizes (figure 3I). For the Early Triassic Chaohu and Nanzhang-Yuan'an faunas, partial disparity was basically provided by Ichthyosauriformes and Sauropterygia, with Ichthyosauriformes occupying the majority (figure 3G). When partial disparity is instead tracked by dental guilds and body sizes, crunch taxa and small species contributed more than half of overall disparity in those Early Triassic faunas (figure 3H,I). The partial disparity reveals several shifts in the Anisian Panxian Fauna and Luoping Biota, where the Eosauropterygia (nothosaurs and pachypleurosaurs) began to contribute more to the overall disparity than ichthyopterygians, accounting for over 50% of disparity (figure 3G). Additionally, crush taxa also began to constitute a large proportion of overall disparity (figure 3H). Taxa with medium to large size (no more than 5 m) replaced taxa with small body size (no more than 1 m) as the dominant contributor to the disparity (figure 3I). For the Ladinian Xingyi Fauna and Carnian Guanling Biota, as shown by partial disparity that was tracked by clades and dental guilds, Sauropterygia and Ichthyosauriformes were still the main contributors to the overall disparity (figure 3G); partial disparity of crush taxa occupied a large proportion, while several other dental guilds shared the remaining part (figure 3H); taxa with body size over 5 m began to contribute to the overall disparity (figure 3I).

## 4. Discussion

### (a) Ecological niche partitioning in South China's Triassic marine reptile faunas

Our ecospace reconstruction reveals five distinct ecotypes among the marine reptiles of South China. Critically, these observed ecotypes did not coexist contemporaneously but emerged sequentially, which reveals a stepwise expansion of ecological functions rather than simultaneous niche filling. Niche partitioning of marine reptiles in each of these Triassic fossil faunas was primarily manifested through spatial and dietary differentiation. Analyses of ecological character distributions reveal that specific traits (including foraging mode, habitat, limb shape and armour type) significantly structured taxon distribution within ecospace (supplementary material, figure S4), showing relationships with habitat partitioning and dietary divergence. These patterns align with broader trends documented in Mesozoic marine tetrapods [31] and conform to Schoener's empirical model of resource partitioning [56]. Even in the Early Triassic Chaohu Fauna, differentiation in dietary strategies and prey preferences is evident: the basal ichthyosauriform *Chaohusaurus* spp. (ecotype 3) possessed superior swimming capabilities and greater aquatic adaptation compared with *Cartorhynchus lenticarpus* (ecotype 1), enabling a more active feeding strategy across broader aquatic habitats [5,57].

This spatial and dietary differentiation intensified over time. Some taxa evolved enhanced aquatic adaptations, enabling ultimately oceanic lifestyles (e.g. derived ichthyosauriforms *Guizhouichthyosaurus*) [55,58]. Concurrently, some other taxa developed durophagous traits to exploit hard-shelled prey (e.g. *Placodus inexpectatus*) [59]. Such divergence reflects a broader trend towards increased functional specialization and more efficient resource usage within increasingly complex ecosystems.

Sympatric species with overlapping functional domains in our ecospace reconstruction likely maintained coexistence through complementarity along other niche axes [56]. Although some taxa overlap in the current ecospace framework, disparity in body size and dental guilds (supplementary material, figure S4) indicates fine-scale resource partitioning. This pattern aligns with Gause's competitive exclusion principle [60], suggesting that niche differentiation along these axes facilitated co-occurrence. Species pairs exhibiting near-complete overlap in habitat or feeding strategy often displayed marked divergence in body size (e.g. small-bodied (<0.5 m) *Cartorhynchus* versus larger medium-sized (1.5–2 m) *Sclerocormus*) [5,19] and/or dental guilds (e.g. *Guizhouichthyosaurus* versus *Guanlingsaurus*) [55]. Such complementarity minimized direct competition for prey resources and promoted community stability, a pattern resembling modern marine ecosystems where tetrapods subdivide ecospace along analogous axes, e.g. body size, foraging depth and diving capacity [14,61,62]. Nevertheless, the inherent limitations of palaeontological data preclude definitive identification of all niche axes. Consequently, the observed coexistence of ecologically proximate species necessitates either fine-scale resource partitioning undetectable through skeletal fossils alone (e.g. underlying microhabitat stratification), or episodic environmental fluctuations maintaining non-equilibrium coexistence [63].

### (b) Community-level ecological dynamics of Triassic marine reptiles in South China

The sequential emergence of the five ecotypes, combined with statistical analyses of ecospace overlap, reveals a cumulative and incremental assembly of Triassic marine reptile ecosystems. With the exception of the two Early Triassic faunas, which differ in habitat setting, ecospace occupation does not show significant differences between temporally adjacent faunas based on statistical tests, yet significant ecospace divergence was detected between temporally distant endpoints (Early Triassic versus Late Triassic faunas). These six faunas can be grouped by geological stage into three distinct phases (figure 4), according to the accompanying trends in ecospace occupation, functional disparity and functional redundancy. All six faunas are exceptionally preserved, and each has been subject to decades of systematic study, so their comparable preservation quality and research history make it unlikely that the temporal trends described below are substantially confounded by differential sampling or preservation [4].

**Figure 4.**
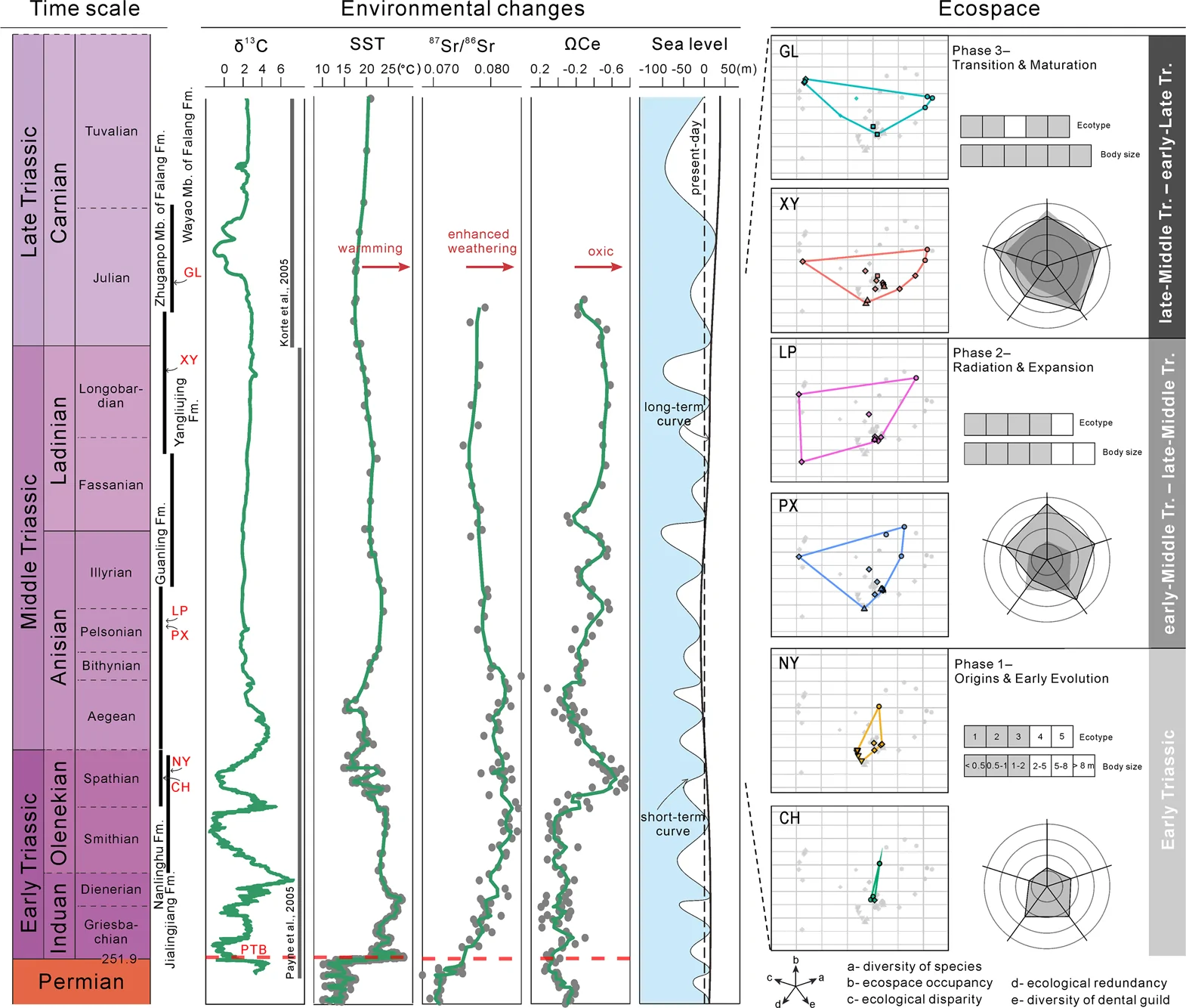
Macroevolutionary phase transitions in community-level ecospace occupancy of Triassic marine ecosystems from the South China basin, mapped against environmental changes. Age assignments of the lithostratigraphic units follow published biostratigraphic, radiometric and cyclostratigraphic constraints (supplementary material). *δ*^13^C data are from Payne *et al.* [15] and Korte *et al.* [64]. Sea surface temperature (SST) is based on conodont *δ*^18^O data from Sun *et al.* [65] and Trotter *et al.* [66]. ^87^Sr/^86^Sr data are from Song *et al.* [67]. Conodont ΩCe data are from Song *et al.* [68]. Sea level changes are modified from [69]. Radar plots show relative values of five ecological metrics. For each ecotype and body-size category, filled boxes indicate categories occupied and open boxes indicate categories absent.

**Phase 1**, spanning from the initial marine invasions of reptiles to the late Early Triassic, is represented by marine reptiles from the Chaohu Fauna and the slightly younger Nanzhang-Yuan'an Fauna [6]. Marine reptile faunas of this phase are characterized by limited ecospace occupation and a low number of functional entities compared with later faunas, with species exhibiting closely aligned ecological niches and high functional redundancy. Most taxa inhabited nearshore shallow environments and exhibited relatively small body sizes; the basal ichthyopterygian *Chaohusaurus*, for instance, rarely exceeded 1 m in total length [57,70]. Despite these shared features, the Chaohu and Nanzhang-Yuan'an faunas show non-overlapping ecospace occupation: both shared ecotypes 1 and 3, but ecotype 2 (armoured ambush carnivores, including Hupehsuchia) was restricted to the latter. This partitioning likely reflects habitat-mediated heterogeneity, as the Chaohu Fauna inhabited a relatively deeper slope setting on the carbonate platform, whereas the Nanzhang-Yuan'an Fauna thrived in a shallow lagoonal environment [6].

**Phase 2**, marked by rapid ecological radiation in the early Middle Triassic, was represented by marine reptiles of the Panxian Fauna and Luoping Biota, both preserved in the Pelsonian (Anisian, Middle Triassic) Guanling Formation [4,21,22]. Faunas of this phase exhibited three features: expanded ecospace occupation, increased ecological disparity, and reduced functional redundancy. These ecological shifts collectively indicate a broader trend towards more specialized and efficient usage of available resources within the ecosystem. Taxonomic diversity markedly increased, with the addition of mixosaurid ichthyopterygians, placodonts, eosauropterygians (nothosaurs, pachypleurosaurs) and archosauromorphs [21,22]. While most non-ichthyopterygian taxa predominantly occupied ecotype 1, reflecting the ecological conservatism [31], expansion into previously unoccupied ecospace (notably subset 2 of ecotype 3 and ecotype 5) reduced potential interspecific competition. This expansion is expressed both in the range of habitats these reptiles could inhabit and in the spectrum of feeding strategies. Such an ecological radiation of marine reptiles was epitomized by their spatial expansions to open oceanic and benthic environments. The body shape of mixosaurid ichthyopterygians approached an optimal morphology for sustained swimming [58,71], making it feasible to swim in open water, corroborated by their wide distribution in both Tethyan and Panthalassic deposits [58]. Concurrently, the number of dental guilds increased, e.g. the unarmoured placodont (*Placodus inexpectatus*) [59] with its crush dental guild facilitated processing hard-shelled benthos. Crush guild taxa began to make up a large proportion of overall disparity (figure 3H). Medium- to large-bodied taxa (< 5 m; e.g. *Nothosaurus*) replace small-bodied taxa as the dominant contributors to the ecological disparity (figure 3I).

**Phase 3** witnessed the ecological transition of Triassic marine reptiles (Ladinian Xingyi Fauna) towards mature, reptile-dominated marine ecosystems (Carnian Guanling Biota) [4,6,21,23,25]. These ecosystems paralleled modern marine tetrapod-dominated ecosystems in trophic structure and trophic complexity [10,11], as recovered by our trait-based ordination (figure 2), exhibiting high taxonomic diversity and significant ecological disparity coupled with resurgence of functional redundancy. The ecological trends initiated in phase 2 (i.e. habitat diversification and trophic specialization), persisted through phase 3, culminating in the marine reptiles’ transition from coastal to open-ocean environments [6,30]. In particular, the full ecological migration of ichthyosauriforms unfolded in three stages: Early Triassic nearshore specialists (ecotype 1, subset 1 of ecotype 3), then early Middle Triassic transitional forms (subset 2 of ecotype 3), and finally Ladinian to Late Triassic pelagic predators (ecotype 4). The ecological roles of Late Triassic ichthyosauriforms converge with those of modern marine mammals (e.g. *Orcinus orca*, *Physeter macrocephalus* and *Stenella attenuata*; figure 2) in ecotype 4 [11,12], highlighting deep-time parallels in apex predator evolution. On the other hand, phase 3 showed an escalation of body size range: megafaunal predators (>5 m; e.g. *Guizhouichthyosaurus* with body size up to 9 m) have commenced their contribution to the overall ecological disparity (figure 3). Direct evidence of this predatory behaviour is preserved in bromalites containing undigested megafaunal remains [55]. Simultaneously, the radiation of armoured taxa (ecotype 5) specialized in exploiting hard-shelled prey may reflect a coevolutionary arms race [72].

### (c) Triassic marine reptiles as ecological innovators—insights from the South China record

The pathways by which marine reptile communities were reassembled after the PTME remain incompletely resolved, in part because trait-based ecospace reconstructions are available for few well-sampled faunas outside a handful of palaeogeographic settings, reflecting the environmentally biased and spatially patchy nature of the Early–Middle Triassic marine tetrapod record [41,73]. The South China succession, which preserves a series of exceptionally preserved marine reptile faunas spanning the Early to Late Triassic, therefore offers a window into the ecological assembly of marine reptile communities over approximately 20 Myr following the extinction [4,6], albeit within a single region.

Across Triassic marine ecosystems generally, marine reptiles acted not merely as ecological ‘replacers’ that refilled niches vacated by the extinction, but as ‘innovators’ [22,74] that restructured marine food webs. Prior to their radiation, some marine ecosystems exhibited an atypical trophic structure, characterized by truncated food chains and accelerated energy flow towards higher trophic levels [17,75]. Several Dienerian and early Spathian assemblages [13,75–78] indicate that fishes temporarily dominated upper trophic levels under these conditions. The subsequent invasion and radiation of marine reptiles altered previous paradigms by introducing new trophic strategies and intensifying predator–prey interactions [13,57,73].

Within the South China record, the initial phase of marine reptile evolution reflects a rapid adaptive radiation into vacant ecological space, but remained ecologically constrained: Early Triassic taxa from South China diversified along a terrestrial-aquatic morphocline [5,20,70,79], exemplifying an early burst pattern, yet remained restricted to nearshore environments [5,20,70]. That role of marine reptiles as ecological innovators became more fully expressed from the Middle Triassic onwards, corresponding to phase 2. The Panxian Fauna and Luoping Biota document structured ecosystems characterized by guild proliferation and reduced functional redundancy [21,22]. Middle to Late Triassic marine reptiles developed a suite of advanced locomotor adaptations (e.g. specialized flippers and streamlined bodies) that supported agile predation in open-ocean settings [40,42]. The rise of pelagic specialists and megafaunal predators plausibly altered the energy transfer and nutrient movement within the marine ecosystems [6,42,57,80]. The extension of air-breathing to open-ocean settings, greater diving depths and substantially larger body sizes have no counterpart among the marine assemblages that preceded them in this basin. We therefore interpret these changes as ecological innovation rather than the refilling of pre-existing niches, and note that the ecospace they occupy (notably ecotype 4) finds its closest analogues not among earlier Triassic assemblages but among modern marine mammals (figure 2) [11].

Environmental stabilization following Early Triassic perturbations was temporally associated with these innovations. Multiple proxies, including carbon isotopes, seawater temperatures, redox conditions and continental weathering rates, indicate a shift from recurrent environmental turmoil to sustained stability beginning in the late Early Triassic (figure 4) [15,64,66–68]. This stabilization, coupled with the recovery of primary productivity [68], may have permitted niche expansion and specialization. Sea level fluctuations may likewise have contributed to the nearshore–offshore transition documented here (figure 4), as such fluctuations correlate with the diversity of shallow marine reptiles and have been implicated in ecological diversification and ecologically selective extinction [14,81]. These associations, however, rest on the temporal correspondence between independent proxy records rather than on direct testing, and other factors such as prey availability and habitat area may also have contributed.

Comparison with assemblages from other palaeogeographic settings, however, indicates that this trajectory in South China was not followed uniformly across the Triassic world. Available records elsewhere show spatial heterogeneity in ecological structure and temporal asynchrony in the appearance of comparable ecological roles [13,57,73]. While macropredatory roles remain poorly documented in the peri-equatorial South China record until the Ladinian [55], at the early Spathian *Grippia* Bonebed of Spitsbergen (approx. 249 Ma) [73,82], the co-occurrence of small-bodied *Grippia* and the giant *Cymbospondylus* indicates that pelagic, megafaunal-scale adaptations (likely equivalent to ecotype 4) had already emerged by the Early Triassic at the Boreal mid-palaeolatitudes [73,83–85]. The giant *Cymbospondylus* (approx. 17 m) [86,87] and *Thalattoarchon* (>8.6 m) [88] from the Anisian of Nevada, along the eastern Panthalassan margin, similarly imply that megafaunal pelagic predators were established there before comparable ecological roles appear in the South China. Several non-mutually-exclusive factors may underlie this asynchrony: contrasting palaeolatitude and thermal regime [65]; differences in depositional setting and habitat segregation prior to Panthalassan dispersal in the late Spathian [57,73]; and the environmentally biased, spatially patchy nature of the Early Triassic tetrapod record itself [13,41,73]. Such heterogeneity and asynchrony are consistent with the contested and structurally complex picture of post-PTME recovery [9,17], and indicate that the described phased trajectory should be read as one of several regionally distinct pathways of ecological assembly.

Against this background of regional variation, the cumulative and incremental assembly documented in South China culminated in pelagic pursuit predators whose ecospace position is comparable to that of modern marine tetrapods, suggesting a long-term conservation of ecological structure at upper trophic levels [12,14]. Notably, marine reptiles in South China achieved broad ecological breadth by the Ladinian, approximately 11 Myr after the PTME, outpacing approximately 20 Myr required for cetaceans to achieve comparable disparity after their Eocene origins [86]. Comparable ecological roles to large pelagic pursuit predators were nonetheless established within approximately 3 Myr after the extinction at Spitsbergen [73,84], further indicating that assembly may have proceeded faster still in some settings. Across these settings, the rapidity of this assembly, together with the ecological novelty discussed above, underscores that marine reptiles were not passive occupants of vacated ecospace but active drivers of ecosystem recovery. This deep-time perspective further argues for protecting extant apex predators and mesopredators as stabilizers of ocean food webs, as they remain disproportionately vulnerable to anthropogenic perturbations [7,14].

## Supplementary Material



## Data Availability

All data and supplementary documents are available in the supplementary material. Supplementary material is available online [89].
