# Supplementary material for: Phased ecological assembly of Triassic marine reptile faunas following the Permian–Triassic mass extinction: ecospace dynamics in South China
Source: Proc Biol Sci. 2026 Oct 14;293(2080):20262108. doi: 10.1098/rspb.2026.2108 (PMC13678723; doi:10.1098/rspb.2026.2108)
Supplement: Supplementary file 1 [file rspb.2026.2108.zip › rspb.2026.2108/rspb20262108_si_001.pdf]

Electronic supplementary material for

Phased ecological assembly of Triassic marine reptile faunas following the Permian-Triassic mass extinction: ecospace dynamics in South China

Mingtao Yao, Yinuo Wang, Zhaoliang Ma, Jiandong Huang, Zuoyu Sun

Proceedings of the Royal Society B: Biological Sciences

Article DOI: 10.1098/rspb.2026.2108

Contents

1. Supplementary figures: S1-S6
2. Supplementary tables: S1-S4
3. Supplementary Text S1: Chronostratigraphic framework of the six South China marine reptile Lagerstätten
4. Supplementary Text S2: Explanation of ecological traits
5. Supplementary Text S3: Brief descriptions for extant marine tetrapods used in this study
6. Supplementary References

1. Supplementary figures

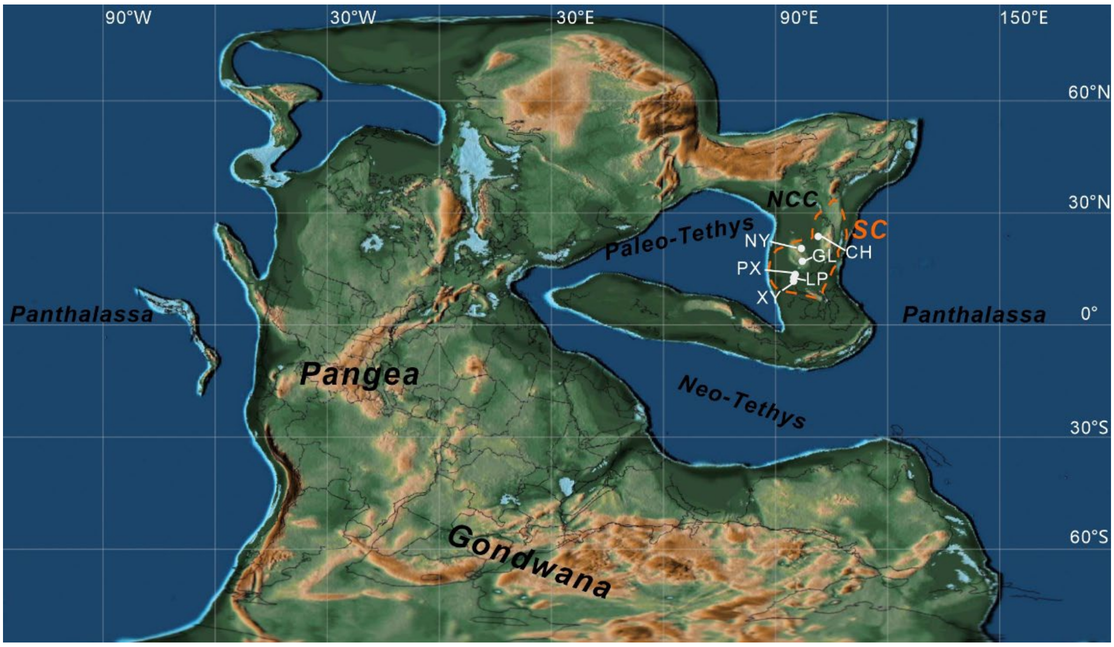

Figure S1. Palaeogeographic position of the six South China marine reptile Lagerstätten analysed in this study. Localities are shown on a Middle Triassic (Anisian, 244 Ma) reconstruction: CH, Chaohu Fauna; NY, Nanzhang-Yuan'an Fauna; PX, Panxian Fauna; LP, Luoping Biota; XY, Xingyi Fauna; GL, Guanling Biota. The approximate extent of the South China Block (SC) is outlined in orange; NCC, North China Craton. Palaeocoordinates were reconstructed in GPlates using the PALEOMAP rotation model, with base map from the PALEOMAP PaleoAtlas for GPlates (Scotese, 2016). Note that reconstructions at this scale do not resolve the platform–basin topography of the

South China carbonate platform, and all localities represent marine depositional settings, as described in the main text.

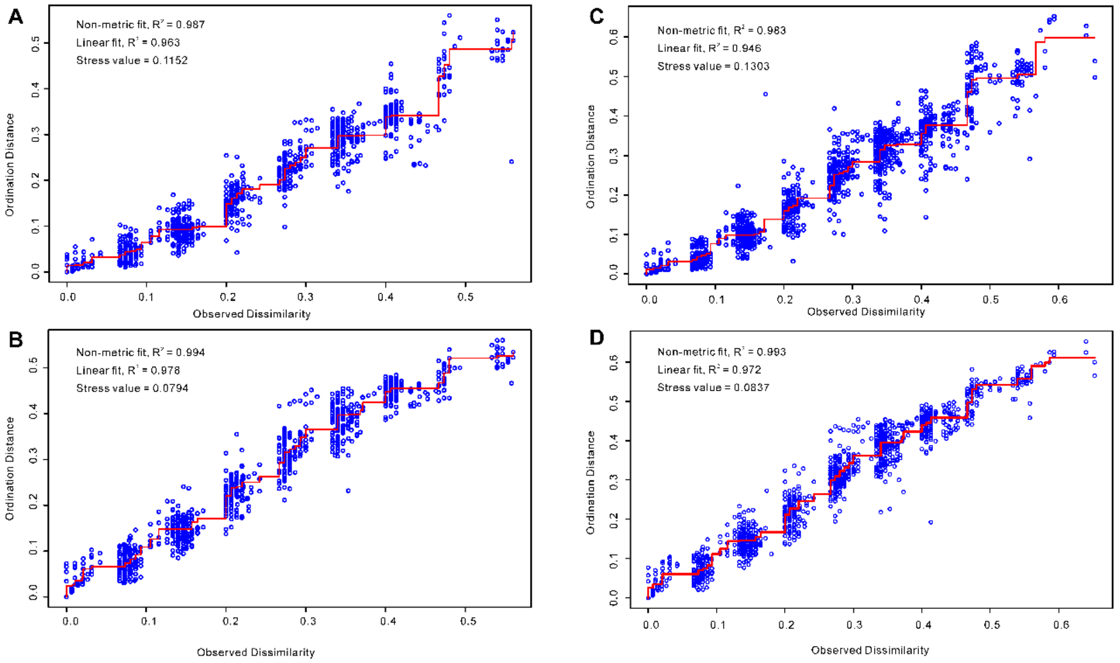

Figure S2. Sensitivity analysis of Non-metric Multidimensional Scaling (NMDS) configurations for ecospace ordination. A. 2D NMDS ordination of the South China Triassic marine reptile fossil dataset, stress value = 0.1152. B. 3D NMDS ordination of the South China Triassic marine reptile fossil dataset, stress value = 0.0794. C. 2D NMDS ordination of extended dataset encompassing marine tetrapods, stress value = 0.1303; D. 3D NMDS ordination of extended dataset encompassing marine tetrapods, stress value = 0.0837.

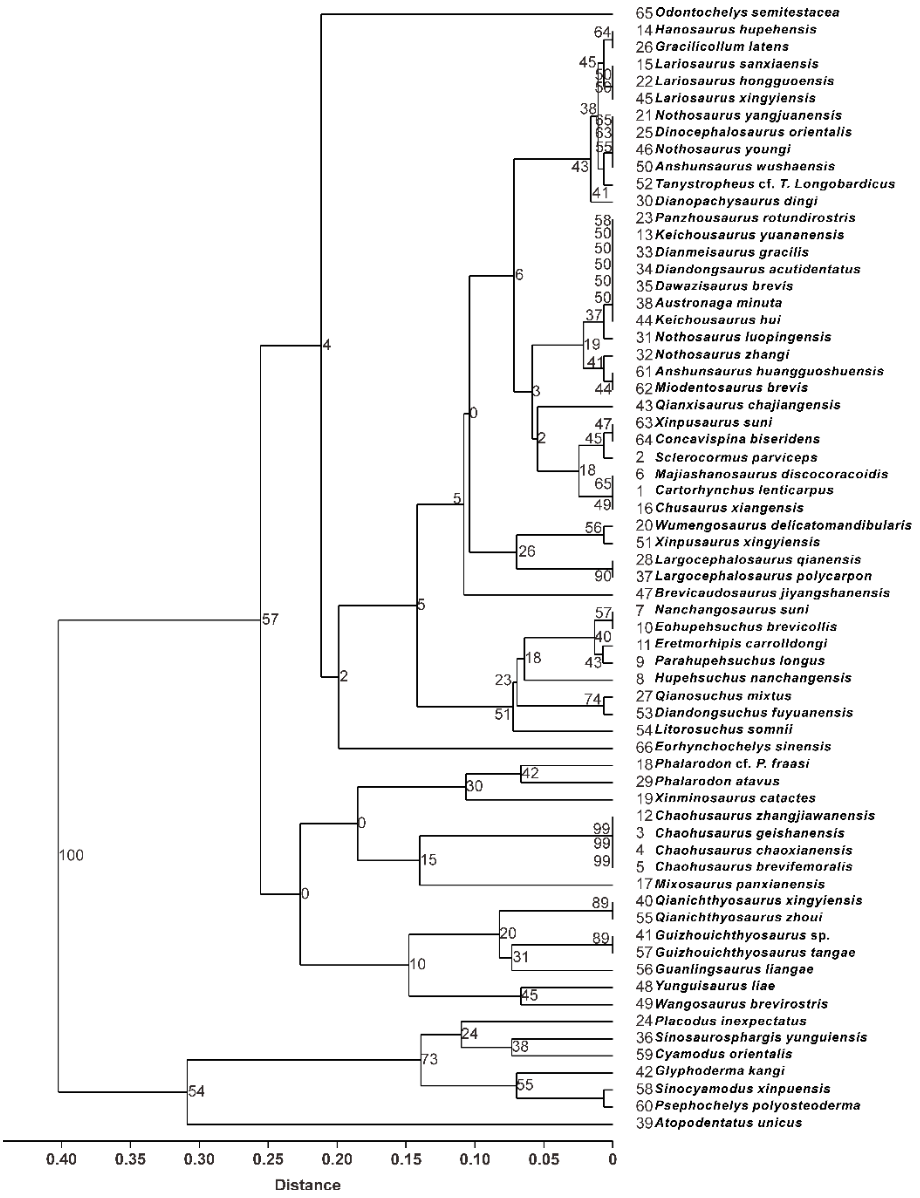

Figure S3. Cluster analysis of fossil dataset based on Gower distance, bootstrap n = 5000.

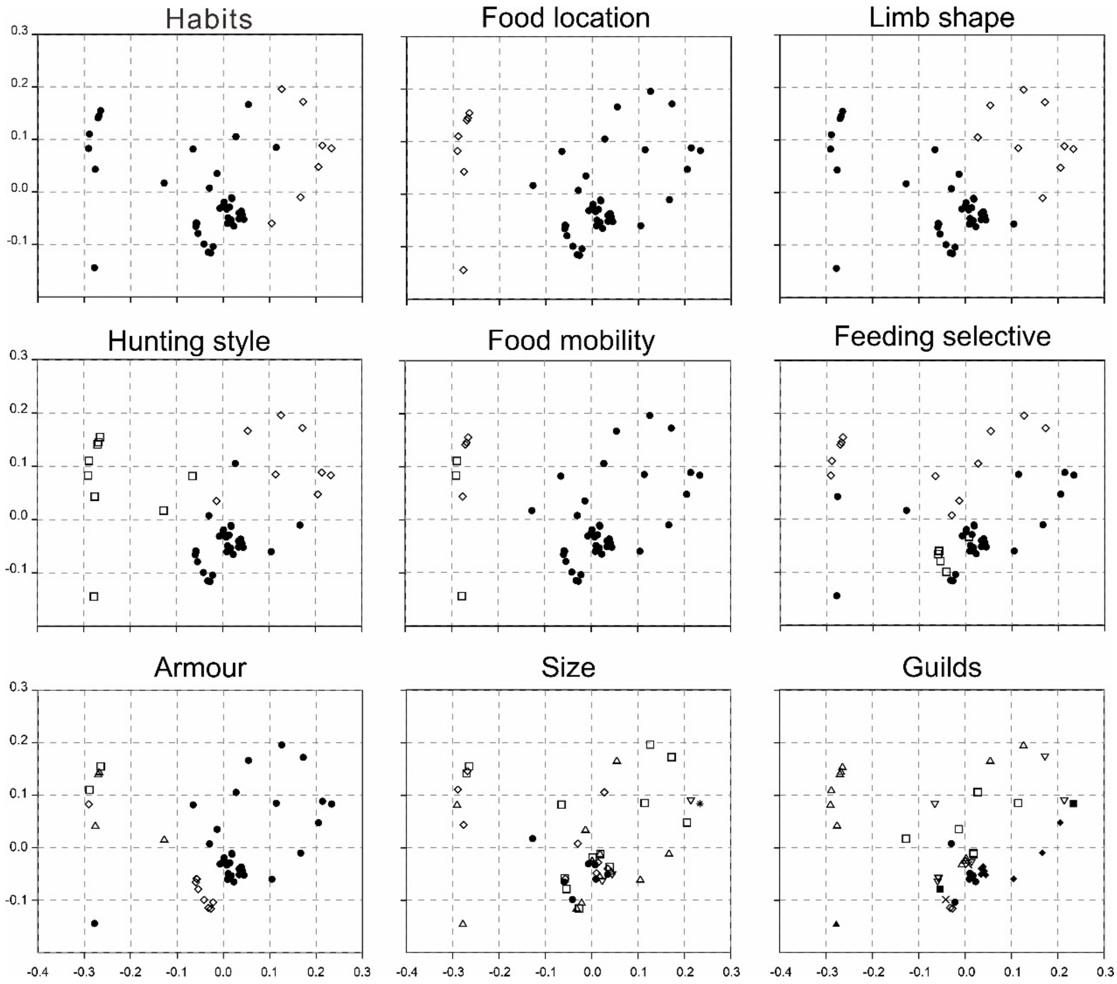

Figure S4. The distribution of ecological character states in 2D ecospace. Each panel illustrates the character scores of individual taxa within ecospace.

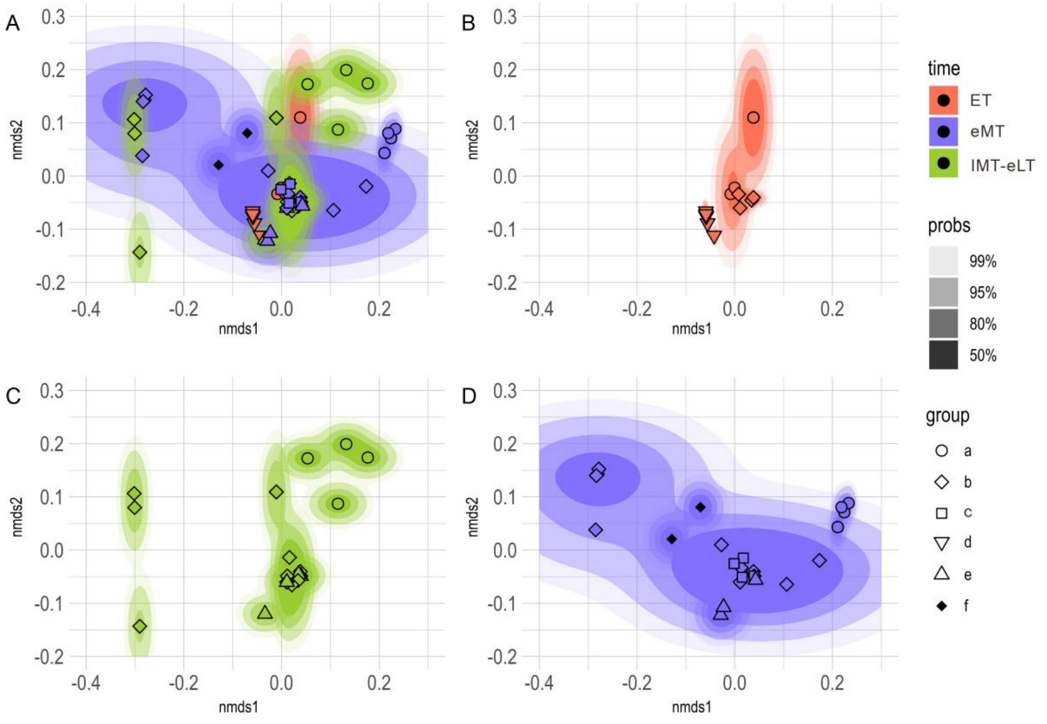

Figure S5. Highest density regions (HDRs) generated via Gaussian kernel smoothing, aiding in visualization of potential spatiotemporal shifts in niche occupation. For abbreviations, Early Triassic (ET), early Middle Triassic (eMT), late Middle Triassic to early Late Triassic (lMT-eLT), Ichthyosauriformes (a), Sauropterygia (b), Thalattosauriformes (c), Hupehsuchia (d), Archosauromorpha (e), Others (d).

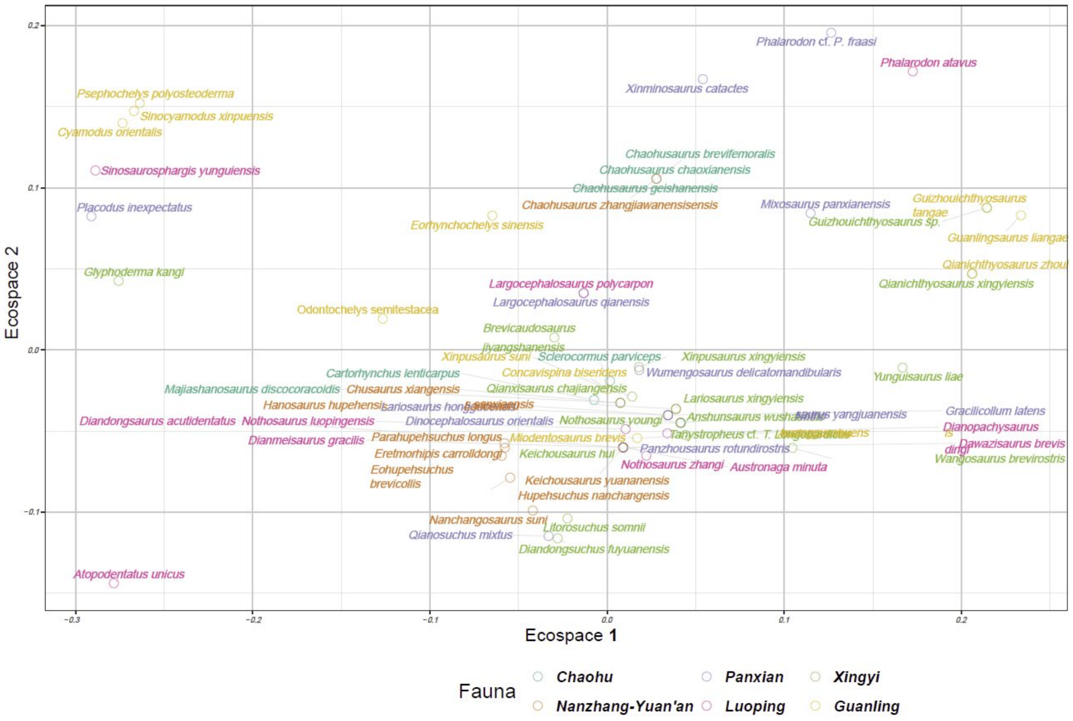

Figure S6. 2D NMDS plot of Triassic marine reptile fossil dataset from South China.

2. Supplementary tables

Supplementary Table S1. Pairwise NP-MANOVA results among ecotypes for the fossil dataset under 2D NMDS configuration. Significance levels: *** $p < 0.001$.

	Ecotype 2	Ecotype 3	Ecotype 4	Ecotype 5
Ecotype 1	$p < 0.001$ ***	$p < 0.001$ ***	$p < 0.001$ ***	$p < 0.001$ ***
Ecotype 2		$p < 0.001$ ***	$p < 0.001$ ***	$p < 0.001$ ***
Ecotype 3			$p < 0.001$ ***	$p < 0.001$ ***
Ecotype 4				$p < 0.001$ ***

Supplementary Table S2. Pairwise NP-MANOVA results among ecotypes for the fossil dataset under 3D NMDS configuration. Significance levels: *** $p < 0.001$.

	Ecotype 2	Ecotype 3	Ecotype 4	Ecotype 5
Ecotype 1	$p < 0.001$ ***	$p < 0.001$ ***	$p < 0.001$ ***	$p < 0.001$ ***
Ecotype 2		$p < 0.001$ ***	$p < 0.001$ ***	$p < 0.001$ ***
Ecotype 3			$p < 0.001$ ***	$p < 0.001$ ***
Ecotype 4				$p < 0.001$ ***

Supplementary Table S3. Pairwise NP-MANOVA results among reptile faunas for the fossil dataset under 2D NMDS configuration. Significance levels: *** $p < 0.001$; ** $p < 0.01$; * $p < 0.05$; ^ $p < 0.1$.

	Nanzhang-Yuan'an	Panxian	Luoping	Xingyi	Guanling
Chaohu	0.0267 *	0.9399	0.3668	0.2441	0.747
Nanzhang-Yuan'an		0.167	0.8569	0.2339	0.1526
Panxian			0.4746	0.4234	0.5835
Luoping				0.288	0.4033
Xingyi					0.1109

Supplementary Table S4. Pairwise NP-MANOVA results among reptile faunas for the fossil dataset under 3D NMDS configuration. Significance levels: *** $p < 0.001$; ** $p < 0.01$; * $p < 0.05$; ^ $p < 0.1$.

	Nanzhang-Yuan'an	Panxian	Luoping	Xingyi	Guanling
Chaohu	0.0384 *	0.6198	0.3219	0.0638 ^	0.0574 ^
Nanzhang-Yuan'an		0.0688 ^	0.2965	0.0302 *	0.0025 **
Panxian			0.8486	0.4287	0.2348
Luoping				0.7396	0.2997
Xingyi					0.0653 ^

3. Supplementary Text S1: Chronostratigraphic framework of the six South China marine reptile Lagerstätten

Chaohu Fauna: The fauna occurs within the Nanlinghu Formation, assigned to the Spathian substage on the basis of detailed stratigraphic study (Zhao et al. 2007; Tong & Zhao 2011). Its age is constrained by ammonoid and conodont biozones, astrochronology and isotopic dating, which together place it in the middle Spathian of the Olenekian, with a zircon U–Pb age of 248.7 ± 1.0 Ma (Ji et al. 2015; Fu et al. 2016; Wang et al. 2025).

Nanzhang-Yuan'an Fauna: The fauna occurs within the Jialingjiang Formation. Subdivision criteria for the members of this formation remain debated, and there is no consensus on whether the fauna should be assigned to Member II or III, although most recent studies place it at the top of Member II (Yan et al. 2021; Wolniewicz et al. 2023; An et al. 2025). Under either scheme, a laterally continuous tuff bed at the top of the fossiliferous horizon provides a reliable marker for age constraint: zircon U–Pb dating has yielded ages of 247.6 ± 1.2 Ma (Cheng et al. 2015) and, most recently, 247.8 ± 1.2 Ma (An et al. 2025). Both are close to the age of the "Green Bean Rock" marking the base of the Middle Triassic in South China, constraining this horizon to the late Spathian.

Panxian Fauna: The fauna occurs in the Upper Member of the Guanling Formation, assigned to the Anisian. Conodont biostratigraphy constrains it to the Pelsonian-Illyrian substages of the Anisian stage (Sun et al. 2006, 2014). Zircon dating of the "red volcanic tuff" bed within the fossiliferous horizon yielded 244.0 ± 1.5 Ma (Wang et al. 2024), consistent with the biostratigraphic age.

Luoping Biota: The biota occurs in the Upper Member of the Guanling Formation, of Anisian age

and closely comparable to that of the Panxian Fauna. Conodont biostratigraphy places it within the Pelsonian substage of the Anisian stage (Huang et al. 2011).

Xingyi Fauna: The fauna occurs in the Zhuganpo Member of the Falang Formation. Its age is Ladinian, supported by ammonoid biostratigraphy (Zou et al. 2015) and isotopic dating (240.8 ± 1.8 Ma) (Li et al. 2016). Cyclostratigraphic analysis further refines the age of the fossiliferous horizon to 240.47–240.30 Ma (Zhang et al. 2026).

Guanling Biota: The biota occurs in the Wayao Member of the Falang Formation, constrained by conodont and ammonoid biostratigraphy (Sun et al. 2003). On the basis of the conodont *Paragondolella auriformis* and the ammonoids *Trachyceras multituberculatum* and *Austrotrachyceras triadicum*, the fauna is not earlier than the Julian (early Carnian) in stage (Sun et al. 2016).

4. Supplementary Text S2: Explanation of ecological traits

Each individual was coded as either binary characters or multistate, ordinal characters. The latter were used only when there was a clear ordination among multiple states. As a result, the present ecospace matrix comprises 29 binary ecological traits, with the exception of ordinal characters for body length.

Binary characters

Diet –This trait is defined by two states, **Carnivore (1)** and **Herbivore (2)** due to inherent limitations in precise paleodietary descriptions of extinct reptiles. Fossil taxa were coded using data from published peer-reviewed studies, while extant taxa followed empirical ecological data. Taxa with potential omnivory were conservatively coded as carnivores to minimize interpretive uncertainty (rather than overinterpretation of limited palaeodietary evidence).

Hunting style – includes **Ambush (3)**, **Pursuit (4)** and **Forage (5)** based on comprehensive assessment of body plan characteristics following established frameworks (Massare, 1988, 1994; Reeves et al., 2020). Ambush predators were identified by either anguilliform swimming mode in axial swimmers or the characteristic paraxial swimming morphology marked by elongated necks for strike range, flexible bodies constrained by armor, and limited limb-driven mobility. Pursuit predators were distinguished by thunniform mode of axial swimmers with sustained swimming adaptations. The foraging predators were reserved for taxa exhibiting minimal aquatic limb/body modifications, predominantly paraxial swimming mode, and morphological indicators of benthic feeding. Fossil taxa were classified using conservative criteria: ambiguous cases were resolved by prioritizing dominant functional morphological traits, consistent with previous applications in Mesozoic marine reptile studies (Reeves et al., 2020). Extant taxa were coded based on observations of their hunting behaviors in modern marine environments.

Feeding guilds – includes **General (6)**, **Cut (7)**, **Crunch (8)**, **Crush (9)**, **Smash (10)**, **Pierce (11)**, **Lunge (12)**, **Filter (13)**, and **Crush-cut (14)** based on comprehensive analysis of dental morphology following established methodologies (Massare, 1987; Motani et al., 2015; Bardet et al., 2015; Foffa et al., 2018). The foundational framework of Massare (1987), as expanded by Foffa et al. (2018), provided the basis for six primary guilds (General to Pierce), while three additional specialized categories were incorporated to accommodate recent discoveries. Lunge feeding was identified in taxa exhibiting prey capture strategies analogous to modern baleen whales (Motani et al., 2015).

The filter guild characterized by numerous slender teeth adapted for bulk feeding. The crush-cut guild was designated for mosasaurs displaying the unique dental morphology described by Bardet et al. (2015). Codes for fossil taxa were systematically reviewed and cross-referenced with the matrix of Reeves et al. (2020).

Feeding selective – includes **Specialist (15)** and **Generalist (16)** for homodont dentition and heterodont dentition, respectively, based on the assumption that those tetrapods with identical (homodont) teeth had a more restricted diet than those with variable (heterodont) tooth shape or size. Fossil taxa were coded based on published literatures, while modern taxa were coded based on current empirical observations on their dentition.

Habitat – includes **Shallow-coastal water (17)** and **Deep open ocean (18)**, reflecting the fundamental distinction between nearshore and pelagic marine environments. This classification was determined primarily by the relative distance from shore and associated ecological characteristics. For fossil taxa, habitat assignments were based on sedimentological analyses of the depositional environments where specimens were preserved, including lithological features and associated fossil assemblages (e.g., benthic invertebrates). Modern taxa were classified according to contemporary ecological observations of their preferred habitats, as documented in marine biology literature and ecological databases

Food location – includes **Pelagic (19)** and **Benthic (20)**, reflecting the vertical distribution of prey resources in marine ecosystems. This simplified classification system was adopted from Bambach's (1983) comprehensive guild framework, which originally recognized six distinct tiering types (pelagic, erect, surficial, semi-infaunal, shallow infaunal, and deep infaunal). It was implemented to accommodate inherent limitations in precisely reconstructing feeding behaviors of extinct marine reptiles. Pelagic designation was applied to taxa targeting prey in the water column, while Benthic classification indicated species foraging on or near the seafloor. Fossil taxa were conservatively assigned as pelagic predators unless morphological evidence (e.g., specialized dentition for durophagy) or associated paleoecological data (e.g., preserved gut contents with benthic organisms) suggested otherwise. Modern taxa were coded based on current empirical observations of their food location.

Food motility – includes **Free moving (21)**, **Sessile (22)** and **Non-sessile (23)**, reflecting differences in prey mobility within marine ecosystems. This classification derives from Bambach's (1983) ecological tiering framework, which originally identified six motility categories, but was simplified here to accommodate uncertainties in reconstructing precise predator-prey dynamics for extinct species. Free moving prey includes actively swimming organisms; Sessile refers to permanently attached prey; while Non-sessile encompasses slow-moving or sedentary but unattached prey. Fossil taxa were coded based on published literatures, while modern taxa were coded based on current empirical observations.

Armour – includes **Non-armoured (24)**, **Osteoderms and scutes (25)**, **Carapace or plastron only (26)**, and **Carapace and plastron (27)**, based on the amount of armour. Heavily armored conditions are interpreted primarily as anti-predator adaptations, while more limited armor may have served additional functions in hydrodynamics or structural support. Fossil taxa were coded based on published peer-reviewed literature. Modern taxa were coded based on direct morphological observations.

Limb shape – includes **Plesiopedal (28)** and **hydropedal (29)** following Reeves et al. (2020), reflecting aquatic adaptation. Plesiopedal was characterized by limbs retaining ancestral terrestrial adaptations, enabling limited land locomotion while maintaining aquatic functionality. Hydropedal was featured by derived aquatic specializations (e.g., hyperphalangy, flattened limb elements) indicating complete commitment to marine life and enhanced capacity for widespread foraging. These states of fossil taxa were partly revised from Reeves et al. (2020). Modern taxa were coded based on empirical ecological data.

Ordered, multistate character

Body length (30) – includes different size-range bins to reflect body size. Standard measurements from rostrum (anterior snout) to tail tip were used to provide a robust and reproducible framework for comparative analyses while accounting for common challenges in paleontological size estimation. For fossil taxa, well-preserved specimens with minimal taphonomic distortion were prioritized, with particular emphasis on adult individuals and type specimens whenever available. Modern taxa were similarly assessed using documented size ranges of adult individuals from ecological literature and museum records, focusing on the typical body length distribution within wild populations. Measurements were primarily obtained from published specimen descriptions of complete skeletons or through direct measurement of illustrated specimens when available. In cases where complete skeletal material was lacking, body length estimates were derived from diagnostic elements (e.g., skull length, vertebral dimensions) using phylogenetically informed allometric scaling relationships with close relatives exhibiting complete preservation. The use of size-range bins rather than single measurements accommodates several sources of biological variation and uncertainty, including ontogenetic growth series, potential taphonomic distortion, and sexual dimorphism documented in some marine reptile groups.

5. Supplementary Text S3: Brief descriptions for extant marine tetrapods used in this study

The **California sea lions (*Zalophus californiancus*)** - a near-shore otariid widely distributed along the coastlines of the North Pacific Ocean. California sea lions are opportunistic predators, primarily consuming a variety of fish (e.g., anchovies, hake, salmon), squids, and occasionally octopuses (Lowry & Maravilla-Chavez, 2005). They often hunt individually or in small groups, utilizing their agility to capture prey in coastal waters and over the continental shelf (Antonelis et al., 1987). California sea lions prefer temperate to tropical coastal waters and are commonly found on sandy beaches, rocky shores, and man-made structures like piers. Adult males are significantly larger than females, reaching lengths of up to 2.4 meters and weights of 275-390 kg, while females typically reach 1.8-2.0 meters and 90-136 kg (Lowry & Maravilla-Chavez, 2005). California sea lions have heterodont dentition, consisting of conical teeth that are well-adapted for grasping and impaling slippery prey like fish and squid. The post-canines are typically single-cusped and sharply pointed, providing an effective tool for seizing fast-moving prey but offering limited masticatory capability. The **northern fur seal (*Callorhinus ursinus*)** - an off-shore otariid that inhabits the open ocean of the North Pacific Ocean and Bering Sea. Northern fur seals are pelagic predators with a diet consisting primarily of small schooling fish (e.g., walleye pollock, capelin), squids, and crustaceans (euphausiids) (Gentry, 1998). They are known for their extensive migratory patterns, traveling thousands of kilometers between their summer breeding rookeries on isolated islands and their

wintering grounds in productive offshore waters (Ream et al., 2005). They typically forage solitarily at night, diving to depths of often 100-200 meters to exploit deep-scattering layers of prey (Gentry, 1998). Northern fur seals exhibit extreme sexual dimorphism. Adult males can reach lengths of 2.1 meters and weigh 180-270 kg, while females are much smaller, averaging 1.4 meters in length and 43-50 kg (Gentry, 1998). Their dentition is heterodont, comprising sharp, conical teeth ideal for capturing and holding soft-bodied pelagic prey.

The **northern elephant seal (e.g., *Mirounga angustirostris*) -** a phocid species found in the North Pacific Ocean. Northern elephant seals are deep-diving predators that feed primarily on mesopelagic fish and squids, including bioluminescent species (Stewart & Huber, 1993). They are renowned for their extreme diving capabilities, regularly reaching depths of 500-800 meters and holding their breath for over 60 minutes, with record dives exceeding 1500 meters and 100 minutes (Le Boeuf et al., 1989). They undertake long, migratory foraging trips lasting several months in the open ocean. In contrast, they aggregate on specific sandy beaches for breeding, molting, and pupping (Stewart & Huber, 1993). This species shows the most pronounced sexual dimorphism of any mammal. Adult bulls can reach 5 meters in length and weigh up to 2700 kg, developing a prominent proboscis, while cows are much smaller, measuring about 3 meters and weighing around 900 kg (Stewart & Huber, 1993). Northern elephant seals possess homodont dentition with 28-30 teeth. The large, conical canines are used primarily as weapons during male-male combat, while the postcanine teeth function to grasp slippery prey.

The **dugong (*Dugong dugon*) -** a species of sirenian that inhabits shallow, tropical and subtropical coastal waters of the Indo-Pacific region, exclusively where seagrass beds are found (Marsh et al., 2011). Dugongs are the only strictly marine herbivorous mammal, feeding almost exclusively on seagrasses. They are suction feeders, using their flexible, bristled snouts to uproot entire plants, including the nutrient-rich roots and rhizomes (Marsh et al., 2011). They are typically solitary or found in small mother-calf pairs, though larger, transient aggregations can occur over abundant seagrass meadows. Dugongs are slow-moving and long-lived, with a low reproductive rate (Marsh et al., 2011). Adults typically measure 2.4-2.7 meters in length and weigh between 250 and 400 kg, with females often being larger than males. The dugong's dentition is highly specialized for its herbivorous diet. Adults lack enamel-crowned incisors and canines. Males and some females possess tusks (first upper incisors) that erupt after sexual maturity, primarily used for social interactions. The molars are open-rooted and continuously grow throughout life to compensate for wear from abrasive seagrass and sediment (Marsh et al., 2011).

The **sea otter (*Enhydra lutris*) -** a marine mustelid native to the northern and eastern coastal regions of the North Pacific Ocean. Sea otters are carnivorous, with a diet that includes a variety of benthic invertebrates such as sea urchins, abalone, clams, and crabs (Riedman & Estes, 1990). They are renowned for their use of tools, frequently using rocks as hammers or anvils to dislodge prey and break open hard shells while floating on their backs. They play a critical ecological role as a keystone species, controlling sea urchin populations which allows kelp forests to thrive (Estes & Palmisano, 1974). Sea otters are primarily found in nearshore habitats with rocky substrates and dense kelp canopies, which provide anchorage and protection from storms (Riedman & Estes, 1990). They are the largest member of the mustelid family but smallest marine mammal. Adults range from 1.2 to 1.5 meters in length and weigh 22-45 kg (Kenyon, 1969). Sea otters possess heterodont dentition, including robust, rounded molars (molariform teeth) that are adapted for crushing the hard exoskeletons of their prey, rather than slicing (Riedman & Estes, 1990).

The **pantropical spotted dolphin** (***Stenella attenuata***) **-** a species of oceanic dolphin that is widely distributed across the tropical and subtropical waters of the world's oceans. Spotted dolphins are opportunistic feeders, primarily consuming small fish, squids, and occasionally crustaceans (Jefferson et al., 2015). These dolphins often hunt in groups, using coordinated efforts to herd schools of fish or squid (Norris and Dohl, 1980). They are known for their acrobatic behavior, which can include leaping out of the water, possibly to spot prey from above or to communicate with other members of their pod. Spotted dolphins prefer warm, deep, offshore waters, but they can also be found in coastal areas (Perrin, 1990). Adult spotted dolphins typically range in length from 1.65 to 2.29 meters and weigh between 85 and 130 kilograms (Jefferson et al., 1993). Spotted dolphins have conical teeth, which are well adapted for grasping slippery prey such as fish and squid (Heyning and Perrin, 1994). The number of teeth can vary, but typically there are between 38 and 44 pairs of teeth in both the upper and lower jaws (Perrin et al., 1981). The teeth are relatively small and closely spaced, providing an effective tool for catching and holding onto fast-moving prey.

The **saltwater crocodile** (***Crocodylus porosus***) **-** found in a wide range of aquatic habitats, including rivers, estuaries, mangrove swamps, and coastal areas. They are highly adaptable and can tolerate both freshwater and saltwater environments. Saltwater crocodiles are apex predators with a highly varied diet that includes fish, crustaceans, birds, reptiles, and mammals. They are ambush predators, using stealth and patience to catch their prey (Lang, 1987). Saltwater crocodiles are the largest living reptiles. Adult males can reach lengths of up to 7 meters and weigh over 1,000 kilograms, although most individuals are smaller, typically ranging from 4 to 5 meters in length. Females are generally smaller, averaging 3 to 3.5 meters in length (Britton, 2010). Saltwater crocodiles have heterodont dentition, meaning their teeth differ in shape and size. They possess large, conical teeth at the front of their jaws, which are used for gripping and holding onto prey. The teeth become progressively smaller toward the back of the jaw, where they are more suited for slicing and crushing (Brochu, 2003).

The **marine iguana** (***Amblyrhynchus cristatus***) **-** primarily herbivorous, feeding almost exclusively on algae that grow on rocks in the intertidal and subtidal zones (Wikelski and Trillmich, 1994). Marine iguanas do not "hunt" in the traditional sense, as their primary food source—algae—is sessile (non-mobile). Instead, they forage by grazing on algae during low tide or while submerged in the water. Marine iguanas are endemic to the Galápagos Islands, an archipelago located in the eastern Pacific Ocean off the coast of Ecuador. They inhabit rocky coastlines, where they can easily access both land and sea (Wikelski and Trillmich, 1997). Marine iguanas exhibit sexual dimorphism, with males being significantly larger than females. Adult males can reach lengths of up to 1.3 meters from snout to tail, while females typically grow to about 0.8 meters (Wikelski et al., 2008). Marine iguanas have homodont dentition, meaning all their teeth are similar in shape and size. Their teeth are trident-shaped, adapted for scraping algae from rocks. Marine iguanas have a form of natural armour in the form of thick, overlapping scales that cover their body. These scales provide protection against abrasion from rocks and potential predators. Additionally, their robust, flattened tail helps them swim efficiently in the water, and their strong limbs with sharp claws allow them to grip rocky surfaces firmly (Wikelski and Trillmich, 1994).

The **green turtle** (***Chelonia mydas***) **-** primarily herbivorous. Their diet consists mainly of seagrasses and algae, which they graze on in shallow coastal waters. However, instead of having true teeth, green turtles have a beak-like structure (tomium) made of keratin (Zug and Vitt, 2012). This beak is sharp and serrated, allowing them to efficiently cut and tear seagrass and scrape algae from surfaces.

Green turtles are one of the largest species of sea turtles. Adult green turtles typically reach lengths of 90–120 cm.

The **Kemp's ridley sea turtle** (***Lepidochelys kempii***) - primarily carnivorous, with a diet that consists mainly of crustaceans, especially blue crabs (Shaver and Caillouet, 1998). They use their powerful, hooked beak and strong jaws to capture and crush the hard shells of their prey. During migrations, Kemp's ridleys may travel long distances across open waters, but they generally stay in coastal or nearshore areas for feeding (Shaver and Caillouet, 1998). Instead of having true teeth, they possess a beak-like structure (tomium) made of keratin. This beak is sharp and hooked, allowing them to efficiently grasp and crush the hard shells of crustaceans and mollusks (Wyneken, 2001). Kemp's ridley turtles are the smallest of all sea turtle species. Adults typically reach 60–70 cm (Zug and Vitt, 2012).

The **sperm whale** (***Physeter macrocephalus***) - apex predators and primarily feed on deep-sea squid, with the giant squid (Architeuthis dux) and colossal squid (Mesonychoteuthis hamiltoni) being notable prey items. The majority of their diet (about 70-90%) consists of squid, particularly those found in the mesopelagic and bathypelagic zones of the ocean (Clarke, 1980). Sperm whales actively chase down squid and other prey in the deep sea. They inhabit deep, pelagic waters in all major oceans, from the tropics to polar regions (Minton et al., 2013). Only the lower jaw is equipped with teeth, while the upper jaw lacks functional teeth. The lower jaw contains between 18 to 26 pairs of conical, peg-like teeth, which are used to grasp and hold slippery prey like squid. The teeth are large, with individual teeth measuring up to 20 cm (8 inches) in length and weighing up to 1 kg (2.2 pounds) each. Despite having teeth, sperm whales do not chew their food; instead, they swallow prey whole or in large pieces (Heyning, 1984). Sperm whales are the largest toothed predators on Earth. Adult males are significantly larger than females, typically ranging from 16 to 20 meters. Length of females are typically 11-13 meters.

The **killer whales** (***Orcinus orca***) - apex predators with a highly varied diet that depends on the population and region, and they can be classified into different ecotypes, feeding on fish and marine mammals (Visser, 2011). Killer whales are found in all oceans and most seas, from tropical to polar regions. Killer whales are highly intelligent and social predators, often hunting in coordinated groups. They possess a set of conical, sharp teeth that are designed for gripping and tearing flesh. Killer whales have a robust, streamlined body shape that allows for efficient swimming at high speeds, which is crucial for chasing fast-moving prey (Williams et al., 2004; Ford and Reeves, 2008). Adult body size varies by sex and population. Males can reach lengths of 6–8 meters. Females range from 5–7 meters in length.

The **walrus** (***Odobenus rosmarus***) - primarily benthic feeders, feeding on organisms that live on or near the seafloor including bivalves, crustaceans and other preys. The primary food sources of walruses—bivalves and other benthic organisms—are sessile or slow-moving and are found in shallow coastal waters (Fay, 1982). Walruses have heterodont dentition. Both males and females have long, curved tusks that grow up to 1 meter in length. They also have premolars and molars that are adapted for crushing the shells of bivalves and other hard prey. The molars are flat and rounded, allowing the walrus to grind down tough, fibrous material (Fay, 1985). Walruses are among the largest pinnipeds (seals, sea lions, and walruses). Males can reach lengths of 3–3.6 meters. Females, typically smaller, can range from 2.5 to 3 meters in length.

6. Supplementary References

An, Z., Fang, Z., Yan, C., Li, J., Liu, Q., Zou, Y., Chen, G., Cheng, L. Stratigraphic framework and timing constraints of Early Triassic Nanzhang-Yuan'an Fauna. Earth Science, 2025;50(7): 2584–2592. (Chinese with English abstract).

Antonelis Jr, G. A., Lowry, M. S., DeMaster, D. P., & Fiscus, C. H. (1987). Assessing northern elephant seal feeding habits by stomach lavage. Marine Mammal Science, 3(4), 308-322.

Bardet, N., Houssaye, A., Vincent, P., Suberbiola, X. P., Amaghzaz, M., Jourani, E., & Meslouh, S. (2015). Mosasaurids (Squamata) from the Maastrichtian phosphates of Morocco: biodiversity, palaeobiogeography and palaeoecology based on tooth morphoguilds. Gondwana Research, 27(3), 1068-1078.

Bambach, R. K. (1983). Ecospace utilization and guilds in marine communities through the Phanerozoic. In Biotic interactions in recent and fossil benthic communities (pp. 719-746). Boston, MA: Springer US.

Boeuf, B. J. L., Naito, Y., Huntley, A. C., & Asaga, T. (1989). Prolonged, continuous, deep diving by northern elephant seals. Canadian Journal of Zoology, 67(10), 2514-2519.

Britton, A. R. C. (2010). Size and growth rate of the saltwater crocodile (Crocodylus porosus). In G. J. W. Webb, C. A. Manolis, & R. W. Stuebing (Eds.), Biology, Evolution, and Conservation of Crocodylians (pp. 101-118). Charles Darwin University Press.

Brochu, C. A. (2003). Phylogenetic approaches toward crocodylian history. Annual Review of Earth and Planetary Sciences, 31, 357-397. https://doi.org/10.1146/annurev.earth.31.100901.141308

Cheng, L., Yan, C., Chen, X., Zeng, X., & Motani, R. (2015). Characteristics and significance of Nanzhang/Yuanan Fauna, Hubei Province. Geology in China, 42(2), 676-684.

Clarke, M. R. (1980). Cephalopod remains from the stomachs of sperm whales caught by the British expeditions in the southern hemisphere. Discovery Reports, 39, 1-96.

Estes, J. A., & Palmisano, J. F. (1974). Sea otters: their role in structuring nearshore communities. Science, 185(4156), 1058-1060.

Fay, F. H. (1982). Ecology and biology of the Pacific walrus, Odobenus rosmarus divergens. North American Fauna, 74, 1-279. https://doi.org/10.3996/nafa.74.0001

Fay, F. H. (1985). Odobenus rosmarus. Mammalian Species, 238, 1-7. https://doi.org/10.2307/3503810

Foffa, D., Young, M. T., Stubbs, T. L., Dexter, K. G., & Brusatte, S. L. (2018). The long-term ecology and evolution of marine reptiles in a Jurassic seaway. Nature Ecology & Evolution, 2(10), 1548-1555.

Ford, J. K. B., & Reeves, R. R. (2008). Fight or flight: Antipredator strategies of baleen whales. Mammal Review, 38(1), 50-86. https://doi.org/10.1111/j.1365-2907.2008.00118.x

Fu, W., Jiang, D. Y., Montañez, I. P., Meyers, S. R., Motani, R., & Tintori, A. (2016). Eccentricity and obliquity paced carbon cycling in the Early Triassic and implications for post-extinction ecosystem recovery. Scientific Reports, 6(1), 27793.

Gentry, R. L. (1998). Behavior and ecology of the northern fur seal (Vol. 382). Princeton University Press.

Heyning, J. E. (1984). Functional morphology of the sperm whale (Physeter catodon), with emphasis on the spermaceti organ and its fluid dynamics. Marine Mammal Science, 1(1), 1-26. https://doi.org/10.1111/j.1748-7692.1985.tb00001.x

Heyning, J. E., & Perrin, W. F. (1994). Evidence for two species of common dolphins (Genus Delphinus) from the eastern North Pacific. Contributions in Science (Natural History Museum

of Los Angeles County), 442, 1-35.
Huang, J., Zhang, K., Zhang, Q., Lü, T., Hu, S., & Zhou, C. (2011). Advance research of conodont fauna from Shangshikan and Daaozi sections in Luoping area, Yunnan Province. Geological Science and Technology Information, 30(3), 1-17.
Jefferson, T. A., Leatherwood, S., & Webber, M. A. (1993). FAO Species Identification Guide: Marine Mammals of the World. Rome: FAO.
Jefferson, T. A., Webber, M. A., & Pitman, R. L. (2015). Marine Mammals of the World: A Comprehensive Guide to Their Identification. Academic Press.
Ji, C., Zhang, C., Jiang, D. Y., Bucher, H., Motani, R., & Tintori, A. (2015). Ammonoid age control of the Early Triassic marine reptiles from Chaohu (South China). Palaeoworld, 24(3), 277-282.
Kenyon, K. W. (1969). The sea otter in the eastern Pacific Ocean (No. 68). US Bureau of Sport Fisheries and Wildlife.
Lang, J. W. (1987). Crocodilian behavior: Implications for management. In G. J. W. Webb, S. C. Manolis, & P. J. Whitehead (Eds.), Wildlife Management: Crocodiles and Alligators (pp. 273-294). Surrey Beatty & Sons.
Li, Z., Sun, Z., Jiang, D., & Ji, C. (2016). LA-ICP-MS Zircon U-Pb age of the fossil layer of Triassic Xingyi Fauna from Xingyi, Guizhou, and its significance. Geological Review, 62(3), 779-790.
Lowry, M. S., & Maravilla-Chavez, O. (2005). Recent abundance of California sea lions in western Baja California, Mexico and the United States. In Proceedings of the sixth California Islands symposium (pp. 485-497). Institute for Wildlife Studies Ventura.
Marsh, H., O'Shea, T. J., & Reynolds, J. E. (2011). Ecology and conservation of the Sirenia: dugongs and manatees (No. 18). Cambridge University Press.
Massare, J. A. (1987). Tooth morphology and prey preference of Mesozoic marine reptiles. Journal of Vertebrate Paleontology, 7(2), 121-137.
Massare, J. A. (1988). Swimming capabilities of Mesozoic marine reptiles: implications for method of predation. Paleobiology, 14(2), 187-205.
Massare, J. A. (1994). Swimming capabilities of Mesozoic marine reptiles. In The mechanics and physiology of animal swimming (pp. 133-147). Cambridge University Press.
Minton, G., Collins, T., Findlay, K. P., & Baldwin, R. (2013). Seasonal distribution and movements of male sperm whales off the Sultanate of Oman. Journal of Mammalogy, 94(3), 640-645. https://doi.org/10.1644/12-MAMM-A-174.1
Motani, R., Chen, X. H., Jiang, D. Y., Cheng, L., Tintori, A., & Rieppel, O. (2015). Lunge feeding in early marine reptiles and fast evolution of marine tetrapod feeding guilds. Scientific reports, 5(1), 8900.
Norris, K. S., & Dohl, T. P. (1980). Behavior of dolphin herds in the Eastern Tropical Pacific Ocean. Fishery Bulletin, 77(4), 891-910.
Perrin, W. F. (1990). Subspecies of Stenella attenuata (Cetacea: Delphinidae). Marine Mammal Science, 6(3), 229-234. https://doi.org/10.1111/j.1748-7692.1990.tb00331.x
Perrin, W. F., Mitchell, E. D., Mead, J. G., Caldwell, D. K., & van Bree, P. J. H. (1981). Stenella clymene, a rediscovered tropical dolphin of the Atlantic. Journal of Mammalogy, 62(3), 583-598. https://doi.org/10.2307/1380405
Ream, R. R., Sterling, J. T., & Loughlin, T. R. (2005). Oceanographic features related to northern fur seal migratory movements. Deep Sea Research Part II: Topical Studies in Oceanography, 52(5-6), 823-843.

Reeves, J. C., Moon, B. C., Benton, M. J., & Stubbs, T. L. (2020). Evolution of ecospace occupancy by Mesozoic marine tetrapods. Palaeontology, 64(1), 31-49.

Riedman, M., & Estes, J. A. (1990). The sea otter (Enhydra lutris): behavior, ecology, and natural history (Vol. 90, No. 14). US Department of the Interior, Fish and Wildlife Service.

Scotese, C.R. (2016). PALEOMAP PaleoAtlas for GPlates and the PaleoData Plotter Program, PALEOMAP Project, http://www.earthbyte.org/paleomap-paleoatlas-for-gplates/ DOI: 10.13140/RG2.2.34367.00166

Shaver, D. J., & Caillouet, C. W. (1998). Diet of juvenile and adult Kemp's ridley sea turtles, Lepidochelys kempii, from Texas, USA. Journal of Herpetology, 32(3), 373-381. https://doi.org/10.2307/1565448

Sun, Z, Hao, W, Jiang, D. (2003) Conodont stratigraphic evidence for the age of the Guanling Fauna, Guizhou Province, China Acta Scientiarum Naturalium Universitatis Pekinensis, 39(1): 118-125. (Chinese with English abstract).

Sun, Z., Sun, Y., Hao, W., & Jiang, D. (2006). Conodont evidence for the age of the Panxian Fauna, Guizhou, China. Acta Geologica Sinica, 80(5), 621-630.

Sun, Z., Jiang, D., Sun, Y., & Hao, W. (2014). Conodont biostratigraphy of the Upper Member of the Guanling Formation in Yangjuan–Chupiwa Section, Guizhou Province, South China. Acta Scientiarum Naturalium Universitatis Pekinensis, 50(2), 269-280.

Sun, Z., Jiang, D., Ji, C., & Hao, W. (2016). Integrated biochronology for Triassic marine vertebrate faunas of Guizhou Province, South China. Journal of Asian Earth Sciences, 118, 101-110.

Tong, J., & Zhao, L. (2011). Lower Triassic and Induan-Olenekian Boundary in Chaohu, Anhui Province, South China. Acta Geologica Sinica-English Edition, 85(2), 399-407.

Visser, I. N. (2011). Predation on thresher (Alopias vulpinus) and sleeper sharks (Somniosus antarcticus) by killer whales (Orcinus orca) specialising on elasmobranch prey. Aquatic Mammals, 37(2), 223-232. https://doi.org/10.1578/AM.37.2.2011.223

Wang Y, Jiang D, Ji C, Sun Z, Zhou M. (2024). Zircon U-Pb age of volcanic tuffs of the Upper Member of the Triassic Guanling Formation and its time constraints for marine reptile fauna in Luxi, Yunnan. Geological Review, 70(5): 2024050037. (Chinese with English abstract)

Wang, Y., Jiang, D., Motani, R., Yao, M., Zhou, M., Sun, Z., Huang, J. & Ma, Z. (2025). Dating of Early Triassic Chaohu Fauna by precise U-Pb age constraints: Temporal calibration of the emergence and first radiation of Mesozoic marine reptiles. Palaeoworld, 200963.

Wikelski, M., & Trillmich, F. (1994). Foraging strategies of the marine iguana, Amblyrhynchus cristatus: Adapting behavioral rules to ontogenetic size change. Behavioral Ecology and Sociobiology, 34(5), 335-344. https://doi.org/10.1007/BF00167760

Wikelski, M., & Trillmich, F. (1997). Body size and individual fitness in marine iguanas. Proceedings of the Royal Society B: Biological Sciences, 264(1387), 1007-1012. https://doi.org/10.1098/rspb.1997.0140

Wikelski, M., Thom, C., Trillmich, F., & Cooke, S. J. (2008). Ontogenetic constraints on thermal preference and swimming performance in the Galápagos marine iguana. Journal of Experimental Biology, 211(11), 1777-1783. https://doi.org/10.1242/jeb.017447

Williams, T. M., Estes, J. A., Doak, D. F., & Springer, A. M. (2004). Killer appetites: Assessing the role of predators in ecological communities. Ecology, 85(12), 3373-3384. https://doi.org/10.1890/03-0696

Wolniewicz, A. S., Shen, Y., Li, Q., Sun, Y., Qiao, Y., Chen, Y., Hu, Y., & Liu, J. (2023). An armoured marine reptile from the Early Triassic of South China and its phylogenetic and evolutionary implications. Elife, 12, e83163.

Wyneken, J. (2001). The Anatomy of Sea Turtles. U.S. Department of Commerce, National Oceanic and Atmospheric Administration, National Marine Fisheries Service.

Yan, C., Li, J., Cheng, L., Zhao, B., Zou, Y., Niu, D., ... & Fang, Z. (2021). Strata characteristics of the Early Triassic Nanzhang-Yuan'an Fauna in Western Hubei Province. Earth Science, 46(1), 122-135.

Zhang, Q., Hu, S., Zhou, C., et al. (2012). Comprehensive Research Report on the Luoping Biota. Chengdu: Chengdu Center of China Geological Survey. (in Chinese).

Zhang, Y., Jiang, D., Motani, R., Tintori, A., Sun, Z., Zhou, M., Su, X., Wang, Y., & Yao, M. (2026). Astronomical age calibration and duration of the Ladinian (Middle Triassic) Xingyi Fauna, southwestern Guizhou Province, China. Palaeogeography, Palaeoclimatology, Palaeoecology, 113652.

Zhao, L., Orchard, M. J., Tong, J., Sun, Z., Zuo, J., Zhang, S., & Yun, A. (2007). Lower Triassic conodont sequence in Chaohu, Anhui Province, China and its global correlation. Palaeogeography, Palaeoclimatology, Palaeoecology, 252(1-2), 24-38.

Zou, X., Balini, M., Jiang, D. Y., Tintori, A., Sun, Z., & Sun, Y. (2015). Ammonoids from the Zhuganpo Member of the Falang Formation at Nimaigu and their relevance for dating the Xingyi fossil-lagerstätte (late Ladinian, Guizhou, China). Rivista Italiana di Paleontologia e Stratigrafia, 121(2).

Zug, G. R., & Vitt, L. J. (2012). Herpetology: An Introductory Biology of Amphibians and Reptiles (4th ed.). Academic Press.
